# Hippo Pathway Activation in Aged Mesenchymal Stem Cells Contributes to the Dysregulation of Hepatic Inflammation in Aged Mice

**DOI:** 10.1002/advs.202300424

**Published:** 2023-08-06

**Authors:** Xue Yang, Chen Zong, Chao Feng, Cangang Zhang, Artem Smirnov, Gangqi Sun, Changchun Shao, Luyao Zhang, Xiaojuan Hou, Wenting Liu, Yan Meng, Liying Zhang, Changshun Shao, Lixin Wei, Gerry Melino, Yufang Shi

**Affiliations:** ^1^ The Third Affiliated Hospital of Soochow University Institutes for Translational Medicine State Key Laboratory of Radiation Medicine and Protection Key Laboratory of Stem Cells and Medical Biomaterials of Jiangsu Province Medical College of Soochow University Soochow University Suzhou 215000 China; ^2^ Department of Experimental Medicine TOR University of Rome Tor Vergata Rome 00133 Italy; ^3^ Department of Tumor Immunology and Gene Therapy Center Third Affiliated Hospital of Naval Medical University Shanghai 200438 China; ^4^ Department of immunology and metabolism National Center for Liver Cancer Shanghai 201805 China; ^5^ Department of Pathogenic Microbiology and Immunology School of Basic Medical Sciences Xi'an Jiaotong University Xi'an Shaanxi 710061 China; ^6^ Department of Clinical Pharmacology The Second Hospital of Anhui Medical University Hefei 230601 China; ^7^ Department of Oncology The First Affiliated Hospital of Anhui Medical University Hefei Anhui 230022 China

**Keywords:** aging‐associated hepatic inflammation, hippo pathway, immunosuppression, mesenchymal stem cells

## Abstract

Aging is always accompanied by chronic diseases which probably attribute to long‐term chronic inflammation in the aging body. Whereas, the mechanism of chronic inflammation in aging body is still obscure. Mesenchymal stem cells (MSCs) are capable of local chemotaxis to sites of inflammation and play a powerful role in immune regulation. Whether degeneration of MSCs in the aging body is associated with unbalanced inflammation is still not clear. In this study, immunosuppressive properties of aged MSCs are found to be repressed. The impaired immunosuppressive function of aged MSCs is associated with lower expression of the Hippo effector Yes‐associated protein 1 (YAP1) and its target gene signal transducer and activator of transcription 1 (STAT1). YAP1 regulates the transcription of STAT1 through binding with its promoter. In conclusion, a novel YAP1/STAT1 axis maintaining immunosuppressive function of MSCs is revealed and impairment of this signal pathway in aged MSCs probably resulted in higher inflammation in aged mice liver.

## Graphical abstract

The decreased immunosuppressive ability of aged mesenchymal stem cells (MSCs) probably results in increased inflammation in aged liver. Hippo pathway activation contributes to the impaired immunosuppressive ability of aged MSCs. Yes‐associated protein 1/signal transducer and activator of transcription 1 axis activation dominates immunosuppression ability of MSCs through inducing iNOS expression upon inflammatory factors stimulation.

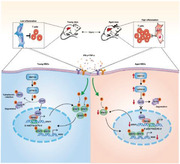

## 1 Introduction

Biological aging is a strong risk factor of many chronic diseases, including osteoarthritis, fibrosis, type II diabetes, Alzheimer's disease, cardiovascular disease, and cancer.^[^ ^1^, ^2^, ^3^, ^4^, ^5^, ^6^, ^7^ ^]^ These age‐related chronic diseases are always caused by long‐term aberrant chronic inflammation, which has been named “inflamm‐aging.”^[^ ^8^ ^]^ Normal levels of inflammation can help organisms to defend against microbial invasion and repair tissues, and the inflammatory response will fade down when it is no longer needed through various immunosuppressive mechanisms.^[^ ^9^, ^10^ ^]^ Unbalanced inflammation could lead to severe organ damage and perturbed homeostasis. In aging organisms, inflammation is difficult to control once triggered. Multiple mechanisms have been reported to contribute to augmentation of age‐related inflammation,^[^ ^11^ ^]^ including redox stress, glycation, dysfunction of mitochondria, deregulation of the immune system, and so on. Whereas, the negative regulation mechanism of inflammation in the “inflamm‐aging” remains unclear and deserves more attention.

Inflamm‐aging is also associated with higher incidence and poor outcomes of liver diseases in the elderly.^[^ ^12^ ^]^ Nonalcoholic fatty liver disease (NAFLD), nonalcoholic steatohepatitis (NASH), alcoholic steatohepatitis (ASH), viral hepatitis, and hepatocellular carcinoma (HCC) were examined more frequently in elderly patients.^[^ ^13^, ^14^, ^15^ ^]^ In this study, we aimed at studying why aged liver is prone to a higher level of inflammation when exposed to injury. Mesenchymal stem cells (MSCs) are a population of adult stem cells that are defined by their ability of self‐renewal and multipotent differentiation.^[^ ^16^ ^]^ MSCs have also been focused on for their nonstem cell properties.^[^ ^17^ ^]^ They are widely considered to be able to migrate into tissue injury sites and inhibit inflammation locally.^[^ ^18^ ^]^ However, aged MSCs have been proved to exhibit an functional decline.^[^ ^19^ ^]^ It is unclear whether immune regulatory dysfunction in aging MSCs results in enhanced age‐related hepatic inflammation and liver diseases. In this study, we will investigate changes in immune regulatory function of aged MSCs and their impact on enhanced age‐related hepatic inflammation. On the other hand, MSCs are also used in clinical trials for the treatment of immune‐related diseases,^[^ ^20^ ^]^ and the functional decline caused by aging limits their clinical application.^[^ ^21^, ^22^, ^23^ ^]^ Thus, this work may also help to build new strategies for improving the clinical efficacy of MSCs.

The hippo signaling pathway was reported to regulate the immune system^[^ ^24^, ^25^ ^]^ and also demonstrated to be dysregulated in aging,^[^ ^26^ ^]^ except for their fundamental functions in tissue development, organ size control, homeostasis, regeneration, and cancer pathogenesis.^[^ ^27^, ^28^, ^29^ ^]^ The active Hippo kinase cascade (which involves the kinases MST1, MST2, LATS1, and LATS2) phosphorylates the core effector Yes‐associated protein 1 (YAP1), which results in its cytoplasmic retention or degradation.^[^ ^30^ ^]^ When the Hippo pathway is shut down, YAP1 translocates into the nucleus and promotes the transcription of target genes. YAP1 has been demonstrated to play a key role in both innate and adaptive immune responses. The role of YAP1 in adaptive immune response was mostly studied in T‐cells of the tumor microenvironment. For instance, YAP1‐induced tumor necrosis factor α (TNF‐α) expression promotes myeloid‐derived suppressor cells and impedes cytotoxic T‐cell infiltration in ovarian carcinomas.^[^ ^31^ ^]^ On the other hand, several studies showed that YAP1 inhibited effector T‐cell differentiation by acting as an internal factor. Gene expression analyses of tumor‐infiltrating T‐cells following *Yap1* deletion implicates YAP1 as a negative mediator of global T‐cell responses in the tumor microenvironment and patient survival in diverse human cancers.^[^ ^32^ ^]^ Therefore, YAP1 can be considered as a negative regulator of T cell response. Overall, the immunoregulatory roles of YAP1 in parenchymal cells, immune cells, and tumor cells have started to be demonstrated.^[^ ^33^, ^34^ ^]^ However, it is still unclear how YAP1 affects the regulation of stromal cells on the adaptive immune response.

In this study, we found that the immunosuppression ability of MSCs declines with aging and this phenomenon is accompanied with YAP1 downregulation. Furthermore, we investigated the underlying mechanisms by which YAP1 regulates immunoregulation of MSCs. This study will help us understand why inflammation in the liver increases with aging and what we can do to improve the clinical efficacy of MSCs in the treatment of immune‐related diseases.

## 2 Results

### 2.1 Liver Inflammation Response Increases with Aging

**Figure 1:**
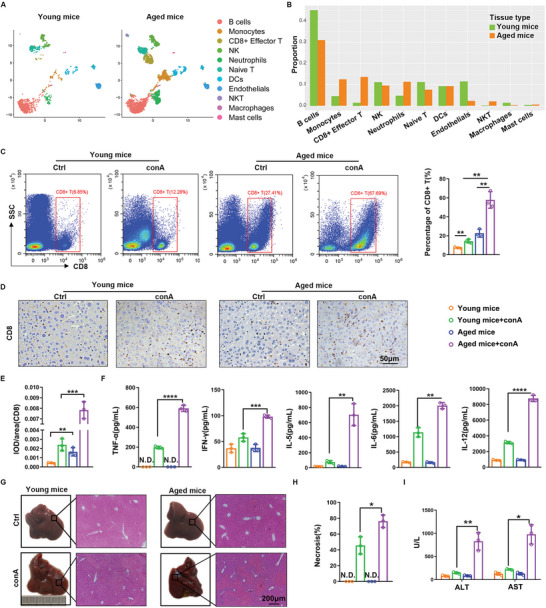
Liver inflammation response increases with aging. A) Previous scRNA‐seq data from liver of young and aged mice were re‐analyzed. Identification of different clusters of cell types in young and aged mouse liver. B) Proportion of different immune cell populations in young and aged mouse liver. C—H) Young and aged mice were intravenously injected with 15 mg kg^‐1^ of conA, followed by sacrificed at 8 h. C, The CD8^+^ T‐cells were subsequently analyzed by FACS and quantified. D, E, IHC staining and quantification of CD8^+^ T‐cells in the liver. F, Concentrations of inflammatory factors in serum were detected by Bio‐plex assay. G, H&E staining of liver sections. H, Quantification of the necrotic area in the liver. I) ALT, and AST were detected in the serum of young and aged mice with or without conA treatment. N.D., not detected. Data were analyzed using two‐tailed unpaired Student *t*‐test. *p < 0.05, **p < 0.01, ***p < 0.001, ****p < 0.0001. Graphs showed mean ± S.D. *N* = 3, the experiments were repeated for three times.

For observing the inflammation level in aged liver, we reanalyzed the data from a previous scRNA‐seq study of liver CD45^+^ immune cells in young (3–6 months) and aged (17–24 months) C57BL/6J mice. Based on the cell markers presented in Figure S2A, Supporting Information, we identified eleven clusters of immune cells (Figure S2B, Supporting Information and **Figure** **1**A) and found that there were more T cells infiltration (CD8^+^ effector T‐cells, naive T‐cells, and NKT cells) in aged liver (23.3% in aged liver &12.5% in young liver) (Figure 1B and Figure S2C). Among the T cells, we can see that CD8^+^ effector T cells were most obviously different between young and aged liver. To further study the alteration of inflammation level, especially CD8^+^ T cell infiltration, in the aged liver with injury, we established a classical autoimmune hepatitis model^[^ ^35^, ^36^, ^37^ ^]^ by using 15 mg kg^−1^ concanavalin A (conA) in young and aged mice, in which CD8^+^ T cells were reported to play main roles at the stage of hepatocyte apoptosis and liver injury.^[^ ^38^ ^]^ Eight hours after conA injection, we quantified total T‐cells by detecting CD3‐positive cells using flow cytometry and immunohistochemistry. The data showed an increased number of infiltrated T‐cells in aged liver of both normal and injured mice (Figure S2D,E, Supporting Information). Then, we analyzed distinct T cell subtypes and several other immune cell types by flow cytometry, including CD8^+^ T cells, CD4^+^ T cells, NK cells, and neutrophils. As shown in Figure 1C, Figure S2F,G, Supporting Information, CD8^+^ T‐cells, and neutrophils responded to conA in both young and aged mice at 8 h after treatment. No difference of neutrophils was observed between conA‐treated young mice and conA‐treated aged mice. However, compared to young mice treated with conA, there was a significantly higher proportion of CD8^+^ T‐cells in conA‐treated aged mice. Immunohistochemical evaluation of CD8 expression also showed that CD8^+^ T‐cells were over‐activated in the aged liver, which is consistent with the results of flow cytometry (Figure 1D,E). To test the inflammatory status of mice, we measured the levels of inflammatory factors in the serum, including TNF‐α, interferon‐γ (IFN‐γ), interleukin 5, interleukin 6, and interleukin 12. Among these cytokines, TNF‐α and IFN‐γ were considered to be critical in conA‐induced liver injury and are associated with activated CD8^+^ T cells.^[^ ^39^, ^40^, ^41^ ^]^ As a result, the serum concentration of inflammatory factors was higher in aged mice (Figure 1F). General observation and H&E staining revealed more severe pathological structural alteration and more hepatic necrosis in aged liver (Figure 1G,H). Serum levels of alanine transaminase (ALT) and aspartate transaminase (AST) were also much higher in aged mice, consistent with liver injury (Figure 1I). These data demonstrated that there is an increased CD8^+^ T‐cell infiltration and higher inflammation in aged mice with conA treatment.

### 2.2 Immunosuppression Capacity of Aged MSCs Decreases

**Figure 2:**
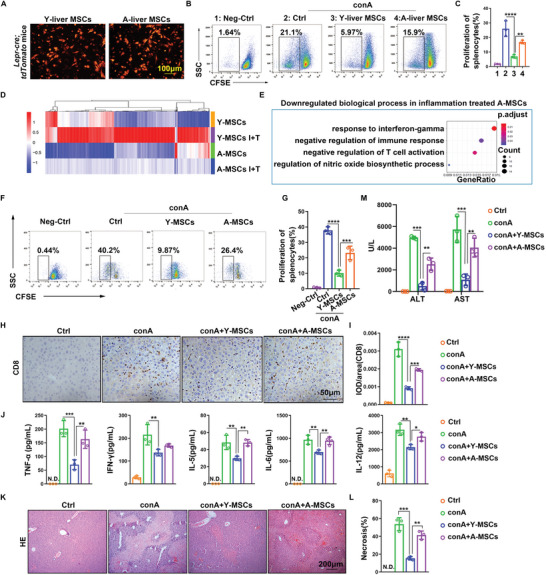
The immunosuppression capacity of aged MSCs is reduced. A) MSCs with tdTomato fluorescence isolated from young and aged *Lepr‐cre*; *loxP‐tdTomato* mice were observed by a fluorescence microscopy. B,C) FACS analysis of the proliferation of splenocytes after co‐culture with young and aged liver MSCs. Splenocytes without treatment were used as negative control. D,E) mRNA expression profiles of young and aged bone marrow MSCs after IFN‐γ plus TNF‐α (I+T) treatment for 2 h were detected by transcriptome sequencing. Heatmap (D) and GO analysis (E) of the differentially expressed genes between young and aged bone marrow MSCs. F,G) FACS analysis and quantification of the proliferation of splenocytes after co‐culture with young and aged bone marrow MSCs. Splenocytes without treatment were used as negative control. H—M) Young and aged bone marrow MSCs (1 × 10^6^ per mouse) were transplanted into young mice at 30 min after injection with 15 mg kg^−1^ of conA. Mice were sacrificed at 8 h after conA injection. Normal young mice were used as control. H, I, IHC staining and quantification of CD8^+^ T‐cells in the liver. J) Levels of inflammatory factors were quantified by Bio‐plex assay in the serum of conA‐treated mice after transplantation of young and aged MSCs. K,L) H&E staining of liver tissue was performed and areas of necrosis were quantified. M) ALT, and AST levels were measured in the serum of mice. Y‐MSCs, young MSCs; A‐MSCs, aged MSCs. N.D., not detected. Data were analyzed using one‐way ANOVA test. *p<0.05, **p < 0.01, ***p < 0.001, ****p < 0.0001. Graphs showed mean ± S.D. *N* = 3, the experiments were repeated for three times.

MSCs have the ability to migrate to injury sites and regulate inflammation locally. To test if uncontrolled inflammation in the aged liver results from degenerative alterations in MSCs, we isolated liver MSCs from young and aged *Lepr‐Cre*; *loxP‐tdTomato mice* upon conA treatment. Leptin Receptor (Lepr) was found to be highly enriched in MSCs^[^ ^42^ ^]^ and thus these *Lepr‐Cre* mice were widely used to track MSCs in vivo.^[^ ^43^, ^44^, ^45^ ^]^ TdTomato‐labeled MSCs from young and aged liver adhered to the bottom of the plate and presented a spindle‐shape morphology (**Figure** **2**A). The liver MSCs were then co‐cultured with CFSE‐labeled splenocytes. Fluorescence activated cell sorter (FACS) analysis showed that aged liver MSCs inhibited the proliferation of splenocytes much less effectively than young liver MSCs (Figure 2B,C).

Previous tracing studies unveiled that MSCs could be recruited from bone marrow upon liver injury， then contribute to liver injury repair and microenvironment improvement via several mechanisms, including multiple differentiation and cytokine secretion.^[^ ^46^, ^47^, ^48^ ^]^ Therefore, we acquired bone marrow MSCs from young and aged WT mice to study the mechanism of weakened immunosuppressive function of aged MSCs. We first characterized their phenotypes and gene expression properties, and aged MSCs expressed more P53 and P21 (Figure S3A,B, Supporting Information). Transcriptome sequencing analysis also showed that the gene expression profile was significantly different between young and aged MSCs (Figure S3C, Supporting Information). Enrichment analysis of the differentially expressed genes showed that aged MSCs expressed higher levels of genes associated with aging‐associated biological processes (Figure S3D, Supporting Information). The inflammatory microenvironment plays an important role in promoting the immunosuppressive effects of MSCs. The combination of IFN‐γ and TNF‐α (I+T) was reported to be able to activate MSCs to suppress inflammation.^[^ ^49^ ^]^ We treated MSCs with I+T inflammatory factors and found that they induced changes of gene expression in both young and aged MSCs (Figure 2D). However, many of the genes which were dramatically upregulated in young MSCs with I+T stimulation were expressed at much lower levels in aged MSCs with I+T stimulation. GO analysis showed that the downregulated genes in I+T‐treated aged MSCs compared with I+T‐treated young MSCs were enriched in processes related to negative regulation of inflammation (Figure 2E). Next, we co‐cultured bone marrow young and aged MSCs with splenocytes to observe the direct immunosuppression effect of MSCs. As shown in Figure 2F,G, we can see that aged MSCs only weakly inhibited splenocyte proliferation.

To further assess the effect of young and aged MSCs on the regulation of inflammation in vivo, we injected young and aged MSCs into conA‐treated young mice with untreated normal young mice as controls. CD8 staining in liver tissue showed that young MSCs greatly reduced the activation of CD8^+^ T cells induced by conA, whereas aged MSCs had a much weaker effect on CD8^+^ T‐cell activation (Figure 2H,I). Detection of inflammatory factors in serum showed that transplantation of young MSCs greatly reduced the levels of inflammatory factors such as TNF‐α and IFN‐γ, but aged MSCs had a weaker effect (Figure 2J). In this liver injury model, conA induced severe hepatocyte necrosis and high levels of the aminotransferases ALT and AST. Transplantation of young MSCs strongly reduced the necrosis and the aminotransferase levels, but the protective effect of aged MSCs was less significant (Figure 2K–M).

The ability of MSCs to suppress inflammation can be affected by various factors, including their survival in vivo, migration, and recruitment to the targeted sites. As YAP1 also plays an important role in regulating cell survival and apoptosis,^[^ ^50^, ^51^ ^]^ we assessed acquisition and apoptosis of young and aged MSCs. As shown in Figure S4A,B, , Supporting Information there were no differences in acquisition between young and aged MSCs on the third and fifth days after isolation. Moreover, the expression levels of apoptosis‐related proteins BIM, BID, and cleaved CASPASE‐3, were comparable between young and aged MSCs. In addition, YAP1 overexpression did not affect apoptosis of aged MSCs (Figure S4C). To verify the migration and homing ability of aged MSCs, we transfected both young and aged MSCs with EGFP‐labeled adenovirus and then transplanted them into mice with liver injury. Recruitment of young and aged MSCs into injured liver was detected by immunofluorescence staining at 8 h posttransplantation. The recruitment of young and aged MSCs to liver injury sites did not exhibit any discernible differences (Figure S4D,E, Supporting Information). We also assessed the migration ability of young and aged MSCs in vitro by transwell assay. The migratory capacity of aged MSCs was comparable to that of young MSCs (Figure S4F,G). Thus, the repressed immunosuppression of aged MSCs is probably attributed to their weakened inhibitory effect on T‐cells, rather than any alterations in survival and migration abilities.

### 2.3 YAP1 is Downregulated in Aged MSCs and Necessary for Immunosuppressive Function of MSCs

**Figure 3:**
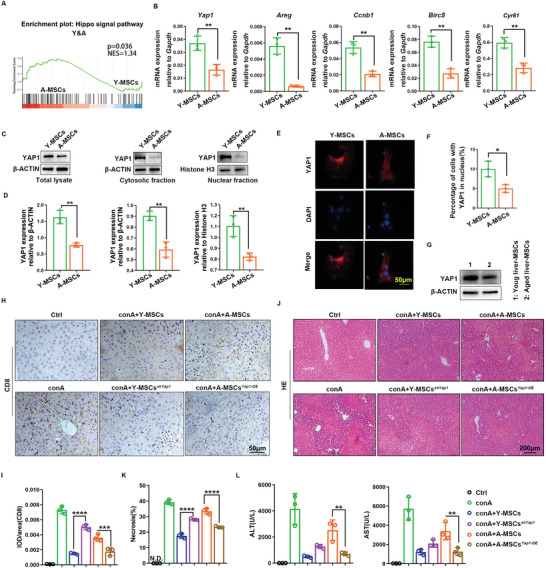
Downregulation of YAP1 in aged MSCs leads to reduced immunosuppressive function. A GSEA analysis of gene expression of young and aged MSCs shows that Hippo signaling is upregulated in aged MSCs. B) mRNA expression levels of *Yap1* and YAP1 target genes were tested by RT‐PCR. C,D) Cytosolic and nuclear expression of YAP1 was detected by western blotting and quantified relative to the internal controls β‐ACTIN and Histone H3. E,F) Translocation of YAP1 into the nucleus was detected in young and aged MSCs by immunofluorescence staining and corresponding quantification. G) YAP1 expression was tested in young and aged liver MSCs by western blotting. β‐ACTIN was used as the internal control. H,I) IHC staining and quantification of CD8^+^ T‐cells in the liver. J,K) H&E staining of the liver after treatment with young and aged MSCs, with or without manipulation of *Yap1* expression, and quantification of the necrotic areas. L) Measurement of serum ALT and AST levels in the indicated mice. Y‐MSCs, young MSCs; A‐MSCs, aged MSCs. N.D., not detected. Data (B, D, F) were analyzed using two‐tailed unpaired Student *t*‐test. Data (I, K, L) were analyzed using one‐way ANOVA test. *p < 0.05, **p < 0.01, ***p < 0.001, ****p < 0.0001. Graphs showed mean ± S.D. *N* = 3, the experiments were repeated for three times.

From GSEA analysis of transcriptome sequencing data from young and aged MSCs, we found that Hippo signaling pathway was upregulated in aged MSCs compared with young MSCs (**Figure** **3**A). We also investigated the activation of MST and LATS kinases of classical Hippo signaling pathway. Hippo signaling was over‐activated in aged MSCs, as indicated by higher expression of LATS1, p‐LATS1/2, MST1/2, and p‐MST1/2 (Figure S5, Supporting Information). The Hippo signaling pathway plays a key role in the immune response which is mainly dependent on the core effector YAP1, which is negatively regulated by Hippo signal activation.^[^ ^25^ ^]^ We then examined the mRNA expression levels of *Yap1* and its target genes (*Areg*, *Ccnb1*, *Birc5* and *Cyr61*) by RT‐PCR. The results showed that all of them were downregulated in aged MSCs (Figure 3B). YAP1 regulates the expression of target genes by translocating to the nucleus and interacting with transcription factors. We observed that nuclear levels of YAP1 were higher in young MSCs than in aged MSCs (Figure 3C–F). We also tested YAP1 expression in young and aged liver MSCs, we found that YAP1 protein levels were indeed downregulated in aged liver MSCs (Figure 3G). On the other hand, we are interested in whether conA could induce YAP1 alteration in liver MSCs and bone marrow MSCs in young and aged mice. We isolated liver and bone marrow MSCs from young and aged mice with and without conA treatment. Western blotting assay showed that conA did not affect YAP1 expression in young and aged MSCs from liver and bone marrow (Figure S6, Supporting Information).

Reverse intervention experiments using adenoviruses were used to verify the role of YAP1 in immunosuppression. *Yap1* was knocked down in young MSCs and overexpressed in aged MSCs, then the modified cells were injected into conA‐treated mice in parallel with control MSCs. The effect of *Yap1* knockdown was verified by western blotting assay. As shown in Figure S7A,B, Supporting Information, *sh1‐Yap1* inhibited the expression of YAP1 and its target genes. As shown in Figure 3H,I, *Yap1* knockdown decreased the ability of young MSCs to suppress CD8^+^ T cells. Artificial supplementation of *Yap1* in aged MSCs rescued the immunosuppression ability of aged MSCs. Detection of inflammatory factors in serum also showed that knockdown of *Yap1* in young and overexpression of *Yap1* in aged MSCs reversed their effect on inflammation (Figure S8, Supporting Information). Knockdown of *Yap1* expression in MSCs also reversed their liver injury repair function (Figure 3J–L). The above data suggest that YAP1 played a key role in the immunosuppressive effect of MSCs, and impaired YAP1 function is probably the main reason for the reduced immunosuppression ability of aged MSCs.

**Figure 4:**
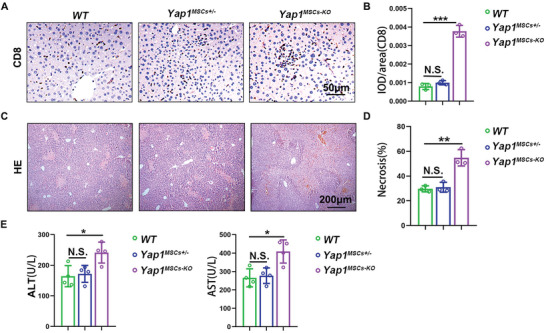
Liver inflammation is increased in conA‐treated *Yap1^MSCs‐KO^* mice. A,B) IHC detection and quantification of activated CD8^+^ T cells in liver of *WT*, *Yap1^MSCs+/‐^* and *Yap1^MSCs‐KO^* mice. C,D) H&E staining of liver of conA‐treated *WT*, *Yap1^MSCs+/‐^* and *Yap1^MSCs‐KO^* mice and quantification of the necrotic area. E) Measurement of serum ALT and AST levels in the indicated mice. Data were analyzed using two‐tailed unpaired Student *t*‐test. *p < 0.05, **p < 0.01, ***p < 0.001. N.S. *p* ≥ 0.05. Graphs showed mean ± S.D. *N* = 3 or 4, the experiments were repeated for three times.

To further verify the role of YAP1 in the immunosuppressive effect of MSCs, we established mice with MSC‐specific knockout (KO) of *Yap1* (*Yap1^MSCs‐KO^*) by crossing *Yap1^FL/FL^* and *Lepr‐cre* mice (Figure S1A,B, Supporting Information). LEPR can be used to trace MSCs in vivo,^[^ ^52^ ^]^ and thus we used it here as a marker of MSCs. *WT*, *Yap1^MSCs+/−^* (heterozygous *Yap1* knockout in MSCs), and *Yap1^MSCs‐KO^* mice were subjected to liver injury by conA injection through the tail vein. As shown in **Figure** **4**A,B, *Yap1* knockout in endogenous MSCs led to more CD8^+^ T cell infiltration. H&E staining and aminotransferase detection showed that *Yap1* knockout in MSCs increased the level of liver injury compared with *WT* mice (Figure 4C–E). Heterozygous *Yap1^MSCs+/−^* MSCs did not show any difference in CD8^+^ T cell infiltration and liver injury compared with *WT* mice. These data suggested that YAP1 played an essential role in the immunosuppression function of MSCs.

### 2.4 YAP1 Maintains the Immunosuppressive Function of MSCs by Promoting iNOS Expression

**Figure 5:**
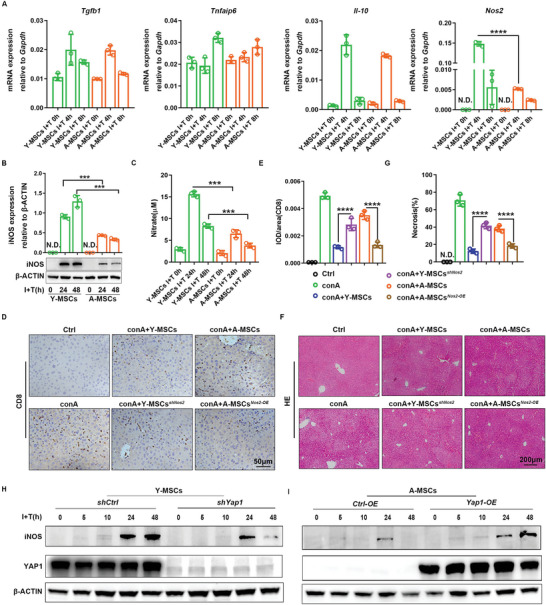
YAP1 maintains the immunosuppressive activity of MSCs by promoting iNOS expression. A) mRNA expression levels of genes encoding anti‐inflammation factors were detected in young and aged MSCs by RT‐PCR after treatment with I+T for 0 h, 4 h, and 8 h. B) Expression of iNOS protein in young and aged MSCs was detected by western blotting assay at 0, 24, and 48 h after I+T treatment and corresponding quantification. β‐ACTIN was used as the internal control. C, Nitrate concentrations were measured by a Griess reagent kit in conditioned medium of young and aged MSCs after I+T treatment. D,E) IHC staining of CD8 positive cells in the liver after treatment with young and aged MSCs (with or without manipulation of *Nos2* expression) and corresponding quantification. F,G) H&E staining of the liver after treatment with young and aged MSCs (with or without manipulation of *No`* expression) and quantification of the necrotic areas. H,I) Young MSCs were transfected by *shYap1* adenovirus, and aged MSCs were transfected by *Yap1* overexpression adenovirus. iNOS expression was detected by western blotting assay in young and aged MSCs treated with I+T for the indicated time. Y‐MSCs, young MSCs; A‐MSCs, aged MSCs. N.D., not detected. Data (A, B, C) were analyzed using two‐tailed unpaired Student *t*‐test. Data (E, G) were analyzed using one‐way ANOVA test. ***p < 0.001, ****p < 0.0001. Graphs showed mean ± S.D. *N* = 3, the experiments were repeated for three times.

Next, we wanted to find the YAP1‐regulated effector molecule which determines the decreased immunosuppressive function of aged MSCs. We analyzed the mRNA expression levels of a series of genes encoding immunosuppression‐related factors in young and aged MSCs after treatment with IFN‐γ and TNF‐α (I+T). The expression of *Nos2* in aged MSCs after I+T treatment was much lower than that in I+T‐treated young MSCs (**Figure** **5**A). Expression of other immunosuppression‐related genes, such as *Tnfaip6*, *Tgfb1*, and *Il10*, was similar between young and aged MSCs (Figure 5A). Analysis of iNOS protein expression and nitrate concentration in the medium of I+T‐treated MSCs verified the above results (Figure 5B,C). Our previous study showed that immunosuppression of MSCs was dependent on iNOS^[^ ^49^ ^]^ and iNOS knockout MSCs had a significantly reduced therapeutic effect on conA‐induced liver injury.^[^ ^53^ ^]^ Further, we verified the effect of iNOS in immunosuppression of young and aged MSCs by reverse intervention experiments in conA‐treated mice. At first, we verified iNOS expression in *Nos2* knockout young MSCs and *Nos2* overexpressing aged MSCs (Figure S7C,D, Supporting Information). As shown in Figure 5D–G, knockout of iNOS weakened the CD8^+^ T‐cell inhibition effect and liver injury repair of young MSCs. Conversely, iNOS overexpression enhanced CD8^+^ T cell inhibition and liver injury repair of aged MSCs. These data indicated that reduction in the level of the immunosuppression factor iNOS probably played a key role in the diminished immunosuppression function of aged MSCs.

Combining the above data, it appears that YAP1 expression is consistent with iNOS expression in young and aged MSCs. Next, we wanted to clarify whether YAP1 regulates iNOS expression. We knocked down *Yap1* in young MSCs and then treated MSCs with IFN‐γ and TNF‐α. iNOS expression was inhibited after *Yap1* knockdown. Overexpression of *Yap1* in aged MSCs enhanced iNOS expression (Figure 5H,I). These data suggested that YAP1 can control iNOS expression.

### 2.5 YAP1 Dominates iNOS Expression via STAT1

**Figure 6:**
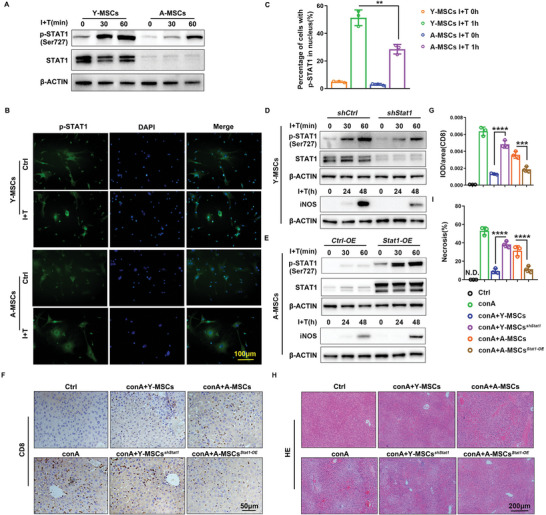
Reduction of iNOS expression in aged MSCs is dependent on STAT1 downregulation. A) Young and aged MSCs were treated with I+T for 0, 30, and 60 min. STAT1 and p‐STAT1 (Ser727) expression were detected by western blotting, β‐actin was used as the internal control. B,C) Immunofluorescence staining and quantification of p‐STAT1 translocation into the nucleus in MSCs after treatment with I+T for 60 min. D) Young MSCs were transfected by *shStat1* adenovirus and then treated with I+T for 0, 30, and 60 min. STAT1 and p‐STAT1 expression were detected by western blotting. β‐ACTIN was used as the internal control. iNOS expression in young MSCs was detected by western blotting after I+T treatment for 0, 24, and 48 h. E) Aged MSCs were transfected by *Stat1* overexpression adenovirus and then treated with I+T for 0, 30, and 60 min. STAT1 and p‐STAT1 expression were detected by western blotting. β‐ACTIN was used as the internal control. iNOS expression in aged MSCs was detected by western blotting after I+T treatment for 0, 24, and 48 h. F, G) IHC staining of CD8 positive cells in the liver after treatment with young and aged MSCs, with or without manipulation of *Stat1* expression and corresponding quantification. H,I) H&E staining of the liver after treatment with young and aged MSCs, with or without manipulation of *Stat1* expression, and quantification of the necrotic areas. Y‐MSCs, young MSCs; A‐MSCs, aged MSCs. N.D., not detected. Data (C) were analyzed using two‐tailed unpaired Student *t*‐test. Data (G, I) were analyzed using one‐way ANOVA test. **p < 0.01, ***p < 0.001, ****p < 0.0001. Graphs showed mean ± S.D. *N* = 3, the experiments were repeated for three times.

The pathways regulating iNOS expression seem to vary in different cells and different species. In general, activation of the transcription factors nuclear factor κB (NF‐κB)) and signal transducer and activator of transcription 1(STAT1) seems to be an essential step in the regulation of iNOS expression in most cells.^[^ ^54^ ^]^ We tested both STAT1 and NF‐κB signaling in young and aged MSCs. As shown in Figure S9A, after I+T stimulation, the expression of p‐P65 in aged MSCs was higher than in young MSCs, and the expression of IκB was lower in aged MSCs. There was also more P65 in the nucleus in aged MSCs with or without I+T treatment (Figure S9B,C, Supporting Information). All of these data indicated that the NF‐κB signal pathway was over‐activated in aged MSCs, which was not consistent with the iNOS expression. We concluded that the diminished immunosuppressive function of aged MSCs was not dependent on NF‐κB signaling alteration. When we analyzed STAT1, we found that both unmodified STAT1 and phosphorylated STAT1 expression were downregulated in aged MSCs (**Figure** **6**A). Furthermore, less p‐STAT1 translocated into the nucleus in aged MSCs after I+T treatment for 60 min (Figure 6B,C). STAT1 is activated by IFN‐γ. Thus, these results could explain why the biological process “response to IFN‐γ” was downregulated in aged MSCs (Figure 2E). Next, we tested iNOS expression after manipulating *Stat1* expression in young and aged MSCs. As shown in Figure S7E, Supporting Information, sh2‐*Stat1* was chosen to knock down the expression of *Stat1*. In young MSCs with *Stat1* knockdown, p‐STAT1 expression, and iNOS expression were both downregulated (Figure 6D). When *Stat1* was overexpressed in aged MSCs, p‐STAT1, and iNOS expression were both upregulated (Figure 6E). These data suggested that decreased expression of STAT1 plays a key role in the decreased iNOS expression in aged MSCs. In consideration of regulation effect of STAT1 on iNOS, we also verified the effect of STAT1 in immunosuppression of MSCs. From Figure 6F–I, we found that reverse intervention of STAT1 in young and aged MSCs reversed their T cell inhibition effect and liver injury repair, which is consistent with iNOS intervention effect as shown in Figure 5D–F.

**Figure 7:**
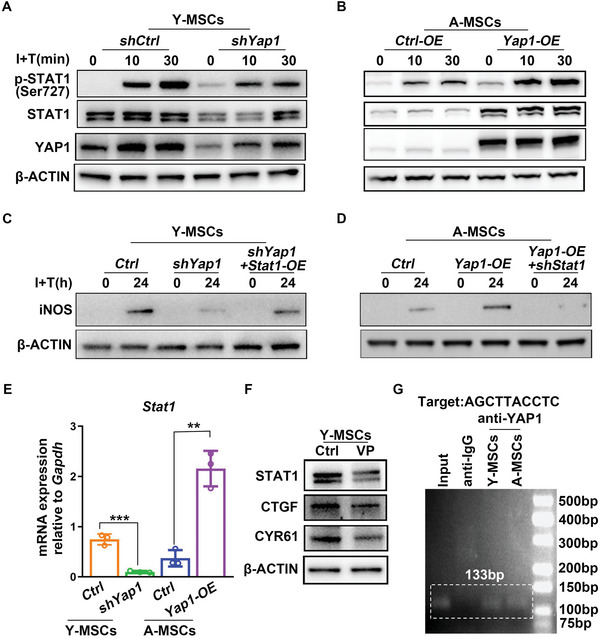
YAP1 promotes iNOS expression by binding the *Stat1* promoter and enhancing *Stat1* transcription. A, B, Young MSCs were transfected by *shYap1* adenovirus and then treated with I+T for 0, 30, and 60 min. Aged MSCs were transfected by *Yap1* overexpression adenovirus and then treated with I+T for 0, 30, and 60 min. Expression of YAP1, STAT1, and p‐STAT1 (Ser727) was detected by western blotting. β‐ACTIN was used as the internal control. C,D) Detection of iNOS expression by western blotting assay in young and aged MSCs in the rescue experiment. β‐ACTIN was used as the internal control. E) The mRNA expression levels of *Stat1* in young and aged MSCs were detected by RT‐PCR after adenovirus‐mediated manipulation of *Yap1* expression. F) Young MSCs were treated with and without 10 µM VP for 24 h. The levels of STAT1, CTGF, and CYR61 were detected by western blotting assay. β‐ACTIN was used as the internal control. G) CUT&Tag assay was performed to verify the target motif bound by YAP1/TEADs in the *Stat1* promoter. Anti‐IgG was used as the negative control. Y‐MSCs, young MSCs; A‐MSCs, aged MSCs. Data were analyzed using two‐tailed unpaired Student *t*‐test. **p < 0.01, ***p < 0.001. The graph showed mean ± S.D. *N* = 3, the experiments were repeated for three times.

To verify the effect of YAP1 on STAT1 expression, we used the *Yap1* intervention adenoviruses to reverse the expression of *Yap1* in young and aged MSCs. Knockdown of *Yap1* in young MSCs suppressed total STAT1 expression and p‐STAT1 expression after I+T treatment (**Figure** **7**A). Overexpression of *Yap1* in aged MSCs promoted STAT1 expression and p‐STAT1 expression after I+T treatment (Figure 7B). To further verify the effect of YAP1/STAT1 signaling in regulating iNOS expression, we performed rescue experiments. *Stat1* overexpression rescued the effect of *Yap1* knockdown in young MSCs, and *Stat1* knockdown reduced the effect of *Yap1* overexpression in aged MSCs (Figure 7C,D). Thus, to conclude, YAP1 regulates iNOS expression through the regulation of STAT1 expression.

### 2.6 YAP1 Regulates STAT1 Expression by Binding the *Stat1* Promoter through YAP1/TEADs Complex

To further elucidate the molecular mechanism underlying the regulation of STAT1 expression by YAP1, we investigated the effect of YAP1 on *Stat1* mRNA expression by RT‐PCR. Knockdown of *Yap1* suppressed the mRNA expression of *Stat1* in young MSCs, and *Yap1* overexpression in aged MSCs promoted the mRNA expression of *Stat1* (Figure 7E). We wanted to understand how YAP1 regulates *Stat1* transcription. Previous studies reported that YAP1, which lacks a DNA‐binding domain, promotes target gene transcription through interaction with TEAD family transcription factors, which bind directly to the promoters or enhancers of target genes.^[^ ^55^, ^56^, ^57^ ^]^ Verteporfin (VP) inhibits the activity of YAP1 by blocking the interaction of YAP1 and TEADs. As shown in Figure 7F, STAT1 expression was suppressed in the presence of VP along with other YAP1 target genes. This result suggested that regulation of STAT1 expression by YAP1 is probably dependent on the binding of YAP1 with TEADs. Through JASPAR, several target sequences of TEADs were predicted in the mouse *Stat1* promoter. To verify molecular mechanism of YAP1/TEADs complex regulating STAT1, we used the CUT&Tag assay upon YAP1/TEADs in both young and aged MSCs. We found that the sequence 5′‐AGCTTACCTC‐3′ in the *Stat1* promoter was bound by YAP1/TEADs complex (Figure 7G).

### 2.7 Higher Expression of YAP1 in MSCs is Correlated with Lower Inflammation in Human Liver Disease

To further explore whether YAP1 expression in MSCs is correlated with the inflammation status in human liver diseases, we analyzed publicly available scRNA‐seq datasets of human liver diseases. Of note, a dataset of human liver fibrosis (GSE186343) contained information related to the inflammation status of the patients. For this dataset, based on cell markers presented in Figure S10A, Supporting Information, we identified 12 cell types (Figure S10B, Supporting Information). MSCs were identified by  four of six marker genes, including *LEPR, CXCL12*, *NES*, *MCAM*, *THY1*, and *NT5E*.^[^ ^58^, ^59^, ^60^, ^61^, ^62^, ^63^ ^]^ Then, we analyzed the correlation between the expression of *YAP1* in MSCs and the inflammation levels in liver. We divided the samples into two groups according to the inflammation levels shown by the authors (Figure S10C, Supporting Information) and we found that samples with higher expression of *YAP1* in MSCs were associated with lower inflammation (Figure S10D, Supporting Information). We also analyzed the correlation of the expression of *YAP1* in MSCs with the ratio of CD8^+^ T‐cells in another four datasets (GSE129933, GSE124395, GSE189903, GSE151530). We integrated these four datasets together and identified thirteen cell types (Figure S11A–C, Supporting Information). Then, we divided the samples into two groups based on *YAP1* expression in MSCs (Figure S11D, Supporting Information) and analyzed the ratio of immune cells. There was a decrease of CD8^+^ T cell proportion in the group with higher expression of *YAP1* in MSCs (Figure S11E, Supporting Information). We also analyzed the correlation of *YAP1* and *STAT1* in human liver MSCs. As shown in Figure S11F, Supporting Information, a strong positive correlation between *YAP1* and *STAT1* was observed.

Altogether, our animal results combined with the scRNA‐seq analysis in human samples suggested that the function of MSCs in suppressing inflammation in liver diseases was dependent on YAP1 expression.

## 3 Discussion

While aging itself is not generally considered as a pathological process, aged liver is more sensitive to injuries and potentially lead to liver diseases. These include NAFLD, NASH, ASH, viral hepatitis, and HCC and occur more frequently in elderly patients.^[^ ^13^, ^14^, ^15^ ^]^ Understanding why the elderly liver is prone to high levels of inflammation is very important for preventing and treating age‐related diseases. Here, we show that downregulation of the YAP1/STAT1 axis activity in aged MSCs resulted in impaired immunosuppression ability of MSCs, and thus contributed to higher inflammation in aged liver.

**Figure 8:**
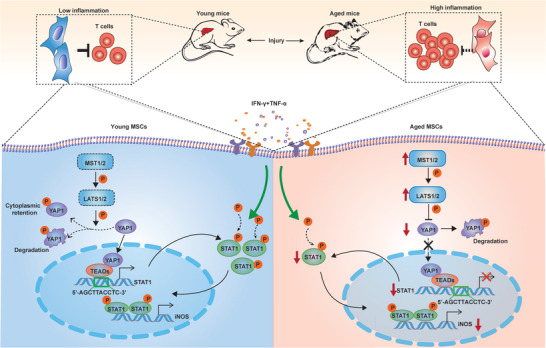
Schematic model to explain the higher level of liver inflammation in aged mouse liver. Higher inflammation in aged mice liver with injury is probably due to immunosuppression impairment of aged MSCs. In young mice, MSCs have an effective inhibitory effect on T‐cells because of higher iNOS expression caused by YAP1/STAT1 axis activation. Whereas, in aged mouse, Hippo signal is over‐activated in MSCs, and iNOS expression driven by YAP1/STAT1 axis is repressed, which leads to impaired inhibitory effect on T‐cells.

First, we analyzed publicly available single‐cell RNA sequencing data carried out in young and aged mouse liver, and found that the proportion of T‐cells, especially CD8^+^ effector T cells, increased significantly in aged mouse liver. CD8^+^ effector T cells, the most common group of effector T‐cells in injured liver, promote inflammation by releasing cytokines and chemokines that recruit other immune cells to the site of infection or tissue damage.^[^ ^64^, ^65^, ^66^ ^]^ These effector T‐cells also directly kill infected or damaged cells through releasing cytotoxic molecules, such as perforin and granzymes. This killing of cells can cause tissue damage and further exacerbate inflammatory responses.^[^ ^67^ ^]^ To investigate the higher inflammation in aged liver and the immune‐regulation role of aged MSCs, we used an inflammatory liver injury model induced by conA. This conA‐based model has been reported to be suitable for investigating the involvement of T‐cell infiltration.^[^ ^68^ ^]^ Our study revealed that aged liver exhibited higher inflammation after injury, and this probably resulted from the repressed immunosuppressive function of aged MSCs targeting CD8^+^ T cells (**Figure** **8**). We also identified a critical role of the YAP1/STAT1 axis in maintaining the immunosuppressive effect of MSCs and the downregulation of YAP1/STAT1 axis probably led to repressed immunosuppression of aged MSCs.

Nonetheless, we also observed a mild increase of other immune cell types in aged liver from the scRNA‐seq analysis (Figure 1B, Supporting Information). Other immune cell types, such as CD4^+^ T cells,^[^ ^69^ ^]^ NK cells,^[^ ^70^ ^]^ and neutrophils^[^ ^71^, ^72^ ^]^ were shown to be involved in conA‐induced liver injury. Therefore, we also tested these immune cells in our model. However, only a significant difference of CD8^+^ T cells between young and aged mice treated with conA was observed. Thus, besides the modulation of CD8^+^ T cells, whether the weakened immunosuppression capability of aged MSCs is also relevant to other immune cell subtypes should be further studied at different stages of conA induced liver injury model.

In previous studies, YAP1 was reported to maintain human MSCs in a younger state, and overexpression of YAP1 rejuvenated senescent MSCs in a replicative senescence model.^[^ ^73^ ^]^ An anti‐aging effect of YAP1 was also found in hepatocytes, hepatic stellate cells, and tumor cells.^[^ ^26^, ^74^, ^75^ ^]^ There are studies demonstrating that YAP1 plays an important role in maintaining the function of stem cells.^[^ ^76^ ^]^ In terms of immune regulation, most studies focused on the role of YAP1 in immune cells and tumor cells. YAP1 can regulate the differentiation of Treg and B cells and the polarity of macrophages.^[^ ^74^ ^]^ There are studies reporting that stromal cells can regulate the tissue microenvironment. Stromal cells have been reported to promote cancer resistance and metastasis.^[^ ^77^, ^78^ ^]^ However, there are few researchers studying the role of YAP1 in immunoregulation in stromal cells. We conducted studies on YAP1‐mediated immunoregulation in MSCs, which are very important stromal cells in the inflammatory microenvironment.

iNOS, one of the most important immunosuppressive factors of MSCs in rodents, was found to be downregulated in aged MSCs, which is due to downregulation of YAP1/STAT1 axis. YAP1 could regulate STAT1 transcription by binding with its promoter through YAP1/TEADs complex. A CUT&Tag experiment using an antibody against YAP1/TEADs helped us to find the exact motif of *Stat1* promoter bound by YAP1/TEADs. We also found Hippo pathway was over‐activated in aged MSCs. However, we are also interested in understanding how the Hippo pathway is regulated in aged MSCs. The Hippo pathway has been reported to be controlled by a variety of mechanisms, including intrinsic cell machineries such as cell‐cell contact, cell polarity, and the actin cytoskeleton molecular cascades, as well as a wide range of extracellular signals, including cellular energy status, mechanical cues, and hormonal signals that act through G‐protein‐coupled receptors (GPCRs).^[^ ^29^ ^]^ GPCR signaling can either activate or inhibit YAP1/TAZ, depending on which classes of downstream heterotrimeric G‐protein are coupled with.^[^ ^79^ ^]^ In addition, data from multiple systems suggested major roles for the GPCR pathways in regulating stem cell function both in vivo and in vitro.^[^ ^79^, ^80^ ^]^ Interestingly, the loss of complexity, sensitivity, and specificity of GPCR signaling over time is likely to be a potent driving factor of pathological aging worldwide.^[^ ^80^ ^]^ Currently, 40% of drugs targeting GPCRs were approved by the FDA^[^ ^81^ ^]^ and have been used in ongoing clinical trials.^[^ ^82^ ^]^ Thus, it would be interesting to conduct further research on the GPCR pathway in aged MSCs.

From the clinical prospective, the role of YAP1 in immune regulation function of human MSCs should be studied further. Using publicly available scRNA‐seq data, we observed a negative correlation between *YAP1* expression in MSCs and inflammation level of patients with liver disease. We also saw a decreased CD8^+^ T‐cells infiltration in patients with higher expression of *YAP1* in MSCs. Our in vivo experiments complemented by the human liver scRNA‐seq analyses suggested that the YAP1/STAT1 axis plays a crucial role in the immunosuppression by MSCs. Therefore, YAP1 overexpression or activation in MSCs could be an attractive strategy to improve MSCs‐based therapies. Currently, several small molecules were identified as potential activators of YAP1 in vitro.^[^ ^83^, ^84^ ^]^ However, further research is needed to develop an efficient platform for YAP1 activation or overexpression in MSCs.

In conclusion, we show a reduced immunosuppressive ability of aged MSCs, which is associated with higher inflammation in aged liver. Mechanistically, we describe a YAP1/STAT1 axis which is important for maintaining immunosuppression properties of MSCs. Therefore, our study provides insight into the mechanism of aging‐associated inflammation and opens new perspectives for improving existing MSCs‐based therapies.

## 4 Experimental Section

### Single‐Cell RNA Sequencing Data Analysis

The data from a previous scRNA‐seq study of CD45^+^ immune cells in young (3–6 months) and aged (17–24 months) C57BL/6 mice were reanalyzed (accession number GEO: GSE155006). The gene expression matrixes of liver immune cells in young and aged mice were imported into Seurat v3^[^ ^85^ ^]^ and merged for subsequent analyses. The following filtering steps were carried out to exclude low‐quality cells: cells with fewer than 300 and more than 5000 detected genes were discarded; cells with a high fraction of mitochondrial genes (>10%) were removed. As a result, a total of 5346 cells with a median of 1187 genes were included in the analyses. Then, nonlinear dimensional reduction was performed with the UMAP (Uniform Manifold Approximation and Projection) method, and cluster biomarkers were found by the “Seurat” package. Furthermore, “ImmGen Datasets” reference database in the Single R package^[^ ^86^ ^]^ was used to annotate the cell type of large cell populations. Procedures used in this study to analyze single cells and to annotate cell types were performed as previously described.^[^ ^87^ ^]^

The human scRNA‐seq dataset (GEO: GSE186343) was from human liver fibrosis with the information of liver inflammation levels. The other four scRNA‐seq datasets (GEO: GSE129933, GSE124395, GSE189903, GSE151530) were from NASH or HCC patients. For all five datasets, the gene expression matrixes were imported into Seurat v4 and merged for subsequent analyses. The following filtering steps were carried out to exclude low‐quality cells: cells with fewer than 200 and more than 7000 detected genes were discarded; cells with a high fraction of mitochondrial genes (>10%) were also removed. “Harmony” package was used to integrate different samples of GSE186343. The other four datasets were integrated together using anchor‐based integration. Then, nonlinear dimensional reduction was performed with the UMAP (Uniform Manifold Approximation and Projection) method, and cluster biomarkers were found by the “Seurat” package. Furthermore, manual annotation was performed based on the specific genes expression in each Seurat cluster.

### Animals

The mice C57BL/6 (6‐8 week‐old, male, weighing 20–22 g) were purchased from the Shanghai Jihui Laboratory Animal Care Co., Ltd. The 6–8 week‐old mice (weighing 20–22 g) were designated as young mice and 20–24 month‐old mice (weighing 30–35 g) were designated as aged mice. *Yap1 ^FL/FL^* mice (C57BL/6 background, 6–8 week‐old) were a gift from Dr. Zhaocai Zhou (Fudan University, China). *Lepr‐Cr*e mice (B6; 129 background, 6–8 week‐old, male, Jackson Lab ID: 008320), *loxP‐tdTomato* mice (C57BL/6 background, 6–8 week‐old, Jackson Lab ID: 007909) and *Nos2^−/‐^* mice (B6; 129P2 background, 6–8 week‐old, male, Jackson Lab ID: 002596) were purchased from JACKSON laboratory. Animals were kept in a specific‐pathogen‐free and temperature‐controlled room with a 12 h dark/light cycle. Experiments were conducted with approval from the Institutional Animal Care and Use Committee of Second Military Medical University (approval number: 20175001123).

### Generation of Yap1^MSCs‐KO^ Mice and Lepr‐cre; loxP‐tdTomato Mice

The floxed Yap1 (*Yap1^FL/FL^*) mice and the mice expressing Cre recombinase under the control of the *Leptin receptor (Lepr)* promoter were used to generate MSC‐specific *Yap1* knockout mice (*Yap1^MSCs‐KO^*). First, a homozygous *Yap1 ^FL/FL^* mouse was mated with a homozygous *Lepr‐cre* mouse to generate F1 mice that were heterozygous for both a loxP‐flanked *Yap1* allele and a *Lepr‐cre* allele (*Yap1^MSCs+/−^*). Next, these F1 mice were backcrossed to the homozygous *Yap1^FL/FL^* mice, resulting in generation of *Yap1^MSCs‐KO^* mice (with a probability of 25%) which were homozygous for the *Yap1 ^FL/FL^* allele and heterozygous for the *Lepr‐cre* allele (Figure S1, Supporting Information). *Lepr‐cre*; *tdTomato* mice were obtained by crossing homozygous *Lepr‐cre mice* with homozygous *loxP‐tdTomato* mice. Double heterozygous F1 mice were used. Mouse genotyping was performed by using a standard PCR protocol with corresponding primers. *Yap1^FL/FL^* was genotyped with the PCR primers: P1, 5′‐CCATTGTCCTCATCTCTTACTAAC‐3′, P2: 5′‐GATTGGGCACTGTCAATTAATGGGCTT‐3′, P3, 5′‐CAGTCTGTAACAACCAGTCAGGGATAC‐3′. *Lepr‐Cre* was genotyped with the PCR primers: P1, 5′‐GCTGGAAGATGGCGATTAGC‐3′, P2, 5′‐CCCAATTTCAAACCTGTTCC‐3′, P3, 5′‐TCTTCTTTCCAGAGTTCAGATGT‐3′. *LoxP‐tdTomato* was genotyped with the PCR primers: P1, 5′‐CGAGGCGGATCACAAGCAATA‐3′, P2, 5′‐TCAATGGGCGGGGGTCGTT‐3′.

### Acute Liver Injury Model

ConA was used to induce autoimmune hepatitis in mice. 3 mg mL^−1^ conA (Vector, California, USA) was injected into mice through the tail vein at a dosage of 15 mg kg^−1^.^[^ ^53^, ^88^ ^]^ Considering the different weights of young and aged mice, the drugs were administered to young and aged mice according to their body weight, aiming to minimize the impact of body weight on drug delivery. Eight hours later, mice were sacrificed and the serum and liver tissues were obtained to test liver injury and inflammation. To compare the therapeutic effect of young and aged MSCs, 1 × 10^6^ MSCs were injected through the tail vein half an hour after conA injection. Five animals were randomly selected for each group.

### Isolation and Culture of Primary Liver MSCs and Bone Marrow MSCs

Male young (6‐8 week‐old) and aged (20‐24 month‐old) *Lepr‐cre*; *loxptdTomato* mice were injected with conA at a dosage of 15 mg/kg. Eight hours later, the livers were removed, cut into pieces, and digested with collagen to obtain single‐cell suspensions.^[^ ^89^ ^]^ Thereafter, nonparenchymal cells were enriched by differential and gradient centrifugation. Then cells were stained with CD29 antibody (1:50, 102216, BioLegend, CA, USA) for 30 min at 4 °C. The cells were washed once with PBS, then diluted to 5 × 10^7^ cells mL^−1^. TdTomato/CD29 double‐positive cells were purified by flow cytometry.

Primary bone marrow MSCs were isolated from 6–8 week‐old and 20–24 month‐old male C57BL/6 mouse bone marrow as described by Zhu et al.^[^ ^90^ ^]^ Young MSCs with *Nos2* knockout were acquired from *Nos2^−/−^* mouse bone marrow. MSCs were cultured with Dulbecco's modified Eagle medium (low glucose) containing 10% fetal bovine serum, 1×glutamax, 1×penicillin‐streptomycin. MSCs after passaging three times were taken as purified MSCs and used to do experiments.

### Flow Cytometry Analysis

Non‐parenchymal cells were isolated from liver as described above, and then diluted to 1 × 10^7^ cells mL^−1^. Suspended cells were stained with CD45 (1:100, 69‐0451‐82, eBioscience, CA, USA), CD3 (1:100, 100347, BioLegend, CA, USA), CD4 (1:100, 404‐0042‐80, eBioscience, CA, USA), CD8 (1:100, 45‐0081‐80, eBioscience, CA, USA), NK1.1 (1:100, 108710, BioLegend, CA, USA), CD11b (1:100, 101208, BioLegend, CA, USA), and Ly6g (1:100, 127617, BioLegend, CA, USA) for 30 min at 4 °C, then washed once in PBS. Cells were then analyzed by flow cytometry.

### Transcriptome Sequencing

The immunosuppressive activity of MSCs could be induced by a combination of IFN‐γ and any of the three proinflammatory cytokines TNF‐α, interleukin 1α, or interleukin 1β.^[^ ^49^ ^]^ In this study, we used IFN‐γ combined with TNF‐α to mimic the inflammatory environment. MSCs were treated with IFN‐γ and TNF‐α for 2 h at a concentration of 10 ng mL^−1^, then control and treated cells were collected and total RNA was extracted by TRIzol (Life Technologies, Grand Island, NY, USA). Sequencing libraries were generated using NEBNext UltraTM RNA Library Prep Kits for Illumina (NEB, USA) following the manufacturer's recommendations by Beijing Biomarker Technologies Co. Ltd. (Beijing, China). Index codes were added to attribute sequences to each sample. Differential expression analysis of two groups was performed using the DESeq2 package. The resulting *P*‐values were adjusted using Benjamini and Hochberg's approach for controlling the false discovery rate. Genes identified by DESeq2 with an adjusted *P* < 0.05 were assigned as differentially expressed.

### Adenovirus‐Mediated Gene Overexpression or Silencing

Adenoviruses were purchased from Obio Technology Corp. Ltd (Shanghai, China). In this study, we used four adenoviruses to overexpress or knock down the target genes *Yap1, Nos2*, and *Stat1*. These adenoviruses were designed using the following procedures. Targeting sequences or scrambled sequences were constructed in the original vectors and these recombinant plasmids were further co‐transfected into HEK293 cells with packaging plasmids. The viral particles in the medium and transfected cells were harvested. For the *Yap1* overexpression adenovirus, the whole CDS of *Yap1* was inserted into the pAdeno‐MCMV‐3Flag‐IRES2‐EGFP vector. For the *Nos2* overexpression adenovirus, the whole CDS of *Nos2* was inserted into the pcADV‐EF1‐mNeonGreen‐CMV‐MCS‐3xFLAG vector. Corresponding empty plasmid was used as control. For the *Yap1* knockdown adenovirus, the shRNA sequence 5′‐AAGCGCTGAGTTCCGAAATCT‐3′ was integrated into the pLKD‐CMV‐EGFP‐2A‐U6‐shRNA vector. 5′‐TTCTCCGAACGTGTCACGT‐3′ was used for scrambled‐shRNA. For the *Stat1* overexpression adenovirus, the whole CDS of *Stat1* was inserted into the pAdeno‐EF1A(S)‐mScarlet‐CMV‐3FLAG vector. Corresponding empty plasmid was used as control. For the *Stat1* knockdown adenovirus, the shRNA sequence 5′‐GCTGAGACTGTTGGTGAAA‐3′ was integrated into the pDKD‐CMV‐mcherry‐U6‐shRNA vector. 5′‐TTCTCCGAACGTGTCACGT‐3′ was used for scrambled‐shRNA.

### Transwell Assay

Transwell assays were performed in 24‐well plates. Twenty thousand MSCs were plated into the upper transwell chamber (8 µm pore) with serum‐free DMEM. DMEM containing 2% FBS was added into the lower chamber. Seventy‐two hours later, cells in the upper inserts were removed and the cells which migrated to the lower surface of the inserts were stained with 0.1% crystal violet.

### Splenocyte Proliferation Assay

Splenocytes were isolated from the spleens of mice by grinding. Freshly isolated splenocytes were stained with 5 µM carboxyfluorescein diacetate succinimidyl ester (CFSE) (eBioscience, California, USA) for 10 min at 37 °C followed by two washes with PBS. CFSE‐labeled splenocytes were co‐cultured with young and aged MSCs separately at a ratio of 10:1 for 72 h in the presence of 5 µg mL^−1^ conA. Then splenocytes were collected and fluorescence was measured by flow cytometry.

### Bio‐Plex Assay

Serum of mice was collected by centrifugation at 3000 rpm for 10 min at 4 °C. Inflammatory cytokines were measured by a Bio‐Plex Pro Mouse Cytokine 23‐plex Assay (Bio‐Rad, California, USA) using Luminex Technology according to the manufacturer's protocol (Bio‐Plex, Bio‐Rad Laboratories).

### Measurement of Nitrate

Young and aged MSCs were treated with IFN‐γ and TNF‐α for 24 h. Cells were cultured for another 24 h after discarding the inflammatory factors. Then the conditioned medium was collected for nitrate detection by using a Griess reagent kit (Beyotime Biotech, Hangzhou, China) according to the manufacturer's instructions.

### RNA Extraction and Real‐Time PCR

Total RNA was extracted from cultured cells by an RNeasy Mini kit (Qiagen, Hilden, Germany) according to the manufacturer's protocol. A Bestar qPCR RT Kit (DBI Bioscience, Ludwigshafen, Germany) was used for reverse transcription to cDNA, and real‐time PCR was performed using a SYBR PrimeScript RT‐PCR Kit (DBI bioscience, Ludwigshafen, Germany) with an ABI Prism 7300 system. The mouse gene primers for real‐time PCR are listed in Table S1, Supporting Information.

### Western Blotting

Cultured cells were lysed with a Minute total protein extraction kit (Invent, Minnesota, USA) with protease inhibitors according to the manufacturer's protocol. Proteins from nucleus and cytoplasm were extracted with a NE‐PER Nuclear and Cytoplasmic Extraction kit (Thermo Fisher Scientific, MA, USA). 20 µg of total protein was loaded for blotting with the indicated primary antibodies. The antibodies were as follows: anti‐iNOS (1:1000, ab49999, Abcam, Cambridge, UK), anti‐P65 (1:1000, 8242, CST, Danvers, MA, USA), anti‐p‐P65 (1:1000, 3033, CST, Danvers, MA, USA), anti‐IκBα (1:1000, 4812, CST, Danvers, MA, USA), anti‐STAT1 (1:1000, 14994, CST, Danvers, MA, USA), anti‐p‐STAT1 (1:1000, 8826, CST, Danvers, MA, USA), anti‐YAP1 (1:1000, ab205270, Abcam, Cambridge, UK), anti‐Histone H3 (1:1000, GB11102, Servicebio, Wuhan, China), anti‐P53 (1:1000, ab131442, Abcam, Cambridge, UK), anti‐P21 (1:1000, ab188224, Abcam, Cambridge, UK), anti‐LATS1 (1:1000, SAB1300096, Merck, Darmstadt, Germany), anti‐p‐LATS1/2 (1:1000, ABS139992, Absin, Shanghai, China), anti‐MST1 (1:1000, ab124787, Abcam, Cambridge, UK), anti‐MST2 (1:1000, 3952, CST, Danvers, MA, USA), anti‐p‐MST1/2 (1:1000, 49332, CST, Danvers, MA, USA), anti‐β‐actin (1:5000, 8457, CST, Danvers, MA, USA), anti‐BID (1:1000, 2003, CST, Danvers, MA, USA), anti‐BIM (1:1000, 2933, CST, Danvers, MA, USA), anti‐CASPASE‐3 (1:1000, 9662, CST, Danvers, MA, USA), and anti‐cleaved CASPASE‐3 (1:1000, 9664, CST, Danvers, MA, USA).

### Immunofluorescence Assay

Cultured cells in 48‐well plates were fixed with 4% paraformaldehyde, followed by permeabilization with 0.25% Triton X‐100 and rinsed with PBS between different steps. To block non‐specific antigen binding, 5% BSA was added for 30 minutes at 37 °C. Primary antibodies were incubated at 4 °C overnight. The next day, cells were rinsed with PBS for three times and incubated with a secondary antibody for 30 min at 37 °C. Cells were rinsed for three times, then DAPI was added for 30 s to stain nuclei. Primary antibodies used were as follows: anti‐YAP1 (1:200, ab205270, Abcam, Cambridge, UK), anti‐P65 (1:200, 8242, CST, Danvers, MA, USA), anti‐STAT1 (1:200, 14994, CST, Danvers, MA, USA), anti‐p‐STAT1 (1:200, 8826, CST, Danvers, MA, USA). Anti‐rabbit secondary antibodies (A11011, A11008, 1:200, Thermo Fisher, USA) conjugated with fluorescent tags were used.

### Hematoxylin and Eosin Staining

Animal tissues were fixed with 4% paraformaldehyde, dehydrated, and embedded in paraffin, then 5‐µm sections were prepared for the experiments. Sections were re‐hydrated and stained using Hematoxylin and Eosin according to the protocol used before.^[^ ^91^ ^]^

### Immunohistochemistry Assay

Procedures were performed as previously described.^[^ ^91^ ^]^ Primary antibodies used in the IHC assay were as follows: anti‐CD3 (1:250, Abcam, Cambridge, UK), anti‐CD8 (1:250, Abcam, Cambridge, UK).

### Rescue Assay

Young MSCs were transfected with *shYap1* adenovirus to inhibit expression of *Yap1*, and 24 h later, *Stat1*‐overexpressing (OE) adenovirus was transfected to enhance *Stat1* expression. Aged MSCs were transfected with *Yap1‐OE* adenovirus to upregulate *Yap1* expression, and 24 h later, *shStat1* adenovirus was transfected to inhibit *Stat1* expression. After 48 h, these cells and non‐treated control cells were all stimulated with IFN‐γ (10 ng mL^−1^) plus TNF‐α (10 ng mL^‐1^) for another 24 h. iNOS was detected by western blotting assay.

### CUT&Tag Assay

The Cleavage Under Target & Tagmentation (CUT&Tag) assay was performed with a NovoNGS CUT&Tag 3.0 High‐Sensitivity Kit (Novoprotein, Soochow, China) according to the manufacturer's instructions. Anti‐IgG antibody (1:50, CST, Danvers, MA, USA), Anti‐YAP1 antibody (1:50, CST, Danvers, MA, USA) was used. The binding sites of TEADs on the *Stat1* promoter were predicted by JASPAR 2020 (http://jaspar.genereg.net). The primers used for target sequence were as follows: forward primer, 5′‐TTTCTCAGTGTAGCCCTGGC‐3′; reverse primer, 5′‐GATCAATTGGGTGCTGATGGC‐3′. The length of PCR product length is 133 bp.

### Statistical Analysis

For animal experiment, three to five mice were randomly chosen for each group. For in vitro assay, three biological replicates were used. All assays were performed more than three times. Data were analyzed with GraphPad Prism 8.0 software using the two‐tailed unpaired Student t‐test and one‐way ANOVA analysis in corresponding experiments. Data were presented as mean ± standard deviation (mean ± S.D.). *p < 0.05, **p < 0.01, ***p < 0.001, ****p < 0.0001 were considered to be significantly different.

## Conflict of Interest

The authors declare no conflict of interest.

## Author Contributions

X.Y., C.Z., and C.F. contributed equally to this work. Y.S., G.M., and L.W. were responsible for the overall concept, design, and supervision of the study. X.Y., C.Z., and C.F. performed the experiments and data analysis. X.Y. wrote the manuscript. C.Z. performed the scRNA‐seq analysis. G.S. and L.Z. helped to culture cells. C.S. revised the paper editing. X.H., W.L., and Y.M. helped to do the animal experiments. L.Z. provided technical support. A.S. and C.S. provided valuable suggestions for data interpretation and manuscript preparation. All authors read and approved the final paper.

## Supporting information

Supporting Information

## Data Availability

The data that support the findings of this study are available from the corresponding author upon reasonable request.

## References

[1] J. C. Pickup , Diabetes Care 2004, 27, 813. 14988310 10.2337/diacare.27.3.813

[2] M. J. Stuart , B. T. Baune , Neurosci. Biobehav. Rev. 2012, 36, 658. 22020230 10.1016/j.neubiorev.2011.10.001

[3] P. Libby , P. M. Ridker , G. K. Hansson , J. Am. Coll. Cardiol. 2009, 54, 2129. 19942084 10.1016/j.jacc.2009.09.009 PMC2834169

[4] R. Wallin , N. Wajih , G. T. Greenwood , D. C. Sane , Med. Res. Rev. 2001, 21, 274. 11410932 10.1002/med.1010

[5] K.‐I. Hirose , H. Tomiyama , R. Okazaki , T. Arai , Y. Koji , G. Zaydun , S. Hori , A. Yamashina , J. Clin. Endocrinol. Metab. 2003, 88, 2573. 12788857 10.1210/jc.2002-021511

[6] S. C. Manolagas , Endocr. Rev. 2000, 21, 115. 10782361 10.1210/edrv.21.2.0395

[7] A. Koyama , J. O'brien , J. Weuve , D. Blacker , A. L. Metti , K. Yaffe , J. Gerontol. A: Biol. Sci. Med. Sci. 2013, 68, 433. 22982688 10.1093/gerona/gls187 PMC3693673

[8] C. Franceschi , M. Capri , D. Monti , S. Giunta , F. Olivieri , F. Sevini , M. P. Panourgia , L. Invidia , L. Celani , M. Scurti , E. Cevenini , G. C. Castellani , S. Salvioli , Mech. Ageing Dev. 2007, 128, 92. 17116321 10.1016/j.mad.2006.11.016

[9] A. Bektas , S. H. Schurman , R. Sen , L. Ferrucci , Exp. Gerontol. 2018, 105, 10. 29275161 10.1016/j.exger.2017.12.015 PMC5909704

[10] M. H. Andersen , Leukemia 2014, 28, 1784. 24691076 10.1038/leu.2014.108

[11] B. Fougère , E. Boulanger , F. Nourhashémi , S. Guyonnet , M. Cesari , J. Gerontol. A: Biol. Sci. Med. Sci. 2017, 72, 1218. 28003373 10.1093/gerona/glw240

[12] A. Floreani , Best Pract. Res. Clin. Gastroenterol. 2009, 23, 909. 19942167 10.1016/j.bpg.2009.10.005

[13] M. Hoare , T. Das , G. Alexander , J. Hepatol. 2010, 53, 950. 20739078 10.1016/j.jhep.2010.06.009

[14] C. Morsiani , M. G. Bacalini , A. Santoro , P. Garagnani , S. Collura , A. D'errico , M. De Eguileor , G. L. Grazi , M. Cescon , C. Franceschi , M. Capri , Ageing Res. Rev. 2019, 51, 24. 30772626 10.1016/j.arr.2019.02.002

[15] E. Cho , H. A. Cho , C. H. Jun , H. J. Kim , S. B. Cho , S. K. Choi , In Vivo 2019, 33, 1411. 31471386 10.21873/invivo.11618 PMC6755010

[16] F. P. Barry , J. M. Murphy , Int. J. Biochem. Cell Biol. 2004, 36, 568. 15010324 10.1016/j.biocel.2003.11.001

[17] A. I. Caplan , Stem Cells Transl. Med. 2017, 6, 1445. 28452204 10.1002/sctm.17-0051 PMC5689741

[18] H. Kawada , J. Fujita , K. Kinjo , Y. Matsuzaki , M. Tsuma , H. Miyatake , Y. Muguruma , K. Tsuboi , Y. Itabashi , Y. Ikeda , S. Ogawa , H. Okano , T. Hotta , K. Ando , K. Fukuda , Blood 2004, 104, 3581. 15297308 10.1182/blood-2004-04-1488

[19] Y. Li , Q. Wu , Y. Wang , L. Li , H. Bu , J. Bao , Int. J. Mol. Med. 2017, 39, 775. 28290609 10.3892/ijmm.2017.2912

[20] X. Yang , Y. Meng , Z. Han , F. Ye , L. Wei , C. Zong , Cell Biosci. 2020, 10, 123. 33117520 10.1186/s13578-020-00480-6 PMC7590738

[21] Y. Wu , J. Yang , Z. Ai , M. Yu , J. Li , S. Li , Gene 2019, 692, 79. 30641220 10.1016/j.gene.2018.12.063

[22] M. S. Choudhery , M. Badowski , A. Muise , J. Pierce , D. T. Harris , J. Transl. Med. 2014, 12, 8. 24397850 10.1186/1479-5876-12-8 PMC3895760

[23] S. Zhou , J. S. Greenberger , M. W. Epperly , J. P. Goff , C. Adler , M. S. Leboff , J. Glowacki , Aging Cell 2008, 7, 335. 18248663 10.1111/j.1474-9726.2008.00377.x PMC2398731

[24] T. Yamauchi , T. Moroishi , Cells 2019, 8, 398. 31052239 10.3390/cells8050398 PMC6563119

[25] L. Hong , X. Li , D. Zhou , J. Geng , L. Chen , Cell Mol. Immunol. 2018, 15, 1003. 29568120 10.1038/s41423-018-0007-1 PMC6269503

[26] Y. T. Yeung , A. Guerrero‐Castilla , M. Cano , M. F. Muñoz , A. Ayala , S. Argüelles , Pharmacol. Res. 2019, 143, 151. 30910741 10.1016/j.phrs.2019.03.018

[27] D. Pan , Dev. Cell 2010, 19, 491. 20951342 10.1016/j.devcel.2010.09.011 PMC3124840

[28] I. M. Moya , G. Halder , Curr. Opin. Cell Biol. 2016, 43, 62. 27592171 10.1016/j.ceb.2016.08.004

[29] F.‐X. Yu , B. Zhao , K.‐L. Guan , Cell 2015, 163, 811. 26544935 10.1016/j.cell.2015.10.044 PMC4638384

[30] L. J. Saucedo , B. A. Edgar , Nat. Rev. Mol. Cell Biol. 2007, 8, 613. 17622252 10.1038/nrm2221

[31] S. Sarkar , C. A. Bristow , P. Dey , K. Rai , R. Perets , A. Ramirez‐Cardenas , S. Malasi , E. Huang‐Hobbs , M. Haemmerle , S. Y. Wu , M. Mcguire , A. Protopopov , S. Jiang , J. F. Liu , M. S. Hirsch , Q. Chang , A. J. Lazar , A. K. Sood , R. Drapkin , R. Depinho , G. Draetta , L. Chin , Genes Dev. 2017, 31, 1109. 28698296 10.1101/gad.296640.117 PMC5538434

[32] E. Stampouloglou , N. Cheng , A. Federico , E. Slaby , S. Monti , G. L. Szeto , X. Varelas , PLoS Biol. 2020, 18, e3000591. 31929526 10.1371/journal.pbio.3000591 PMC6980695

[33] B. Liu , Y. Zheng , F. Yin , J. Yu , N. Silverman , D. Pan , Cell 2016, 164, 406. 26824654 10.1016/j.cell.2015.12.029 PMC4733248

[34] V. Ramjee , D. Li , L. J. Manderfield , F. Liu , K. A. Engleka , H. Aghajanian , C. B. Rodell , W. Lu , V. Ho , T. Wang , L. Li , A. Singh , D. M. Cibi , J. A. Burdick , M. K. Singh , R. Jain , J. A. Epstein , J. Clin. Invest. 2017, 127, 899. 28165342 10.1172/JCI88759 PMC5330722

[35] S. Yan , L. Wang , N. Liu , Y. Wang , Y. Chu , Immunol. Cell Biol. 2012, 90, 421. 21691280 10.1038/icb.2011.59

[36] X.‐J. Ye , R. Xu , S.‐Y. Liu , B. Hu , Z.‐J. Shi , F.‐L. Shi , B. Zeng , L.‐H. Xu , Y.‐T. Huang , M.‐Y. Chen , Q.‐B. Zha , X.‐H. He , D.‐Y. Ouyang , Int. Immunopharmacol. 2022, 102, 108380. 34848154 10.1016/j.intimp.2021.108380

[37] Y. Liu , H. Hao , T. Hou , Open Life Sci. 2022, 17, 91. 35291566 10.1515/biol-2022-0013 PMC8886606

[38] F. Heymann , K. Hamesch , R. Weiskirchen , F. Tacke , Lab. Anim. 2015, 49, 12. 25835734 10.1177/0023677215572841

[39] L. Dara , Z.‐X. Liu , N. Kaplowitz , J. Clin. Invest. 2016, 126, 4068. 27760053 10.1172/JCI90830 PMC5096892

[40] S. Kusters , F. Gantner , G. Kunstle , G. Tiegs , Gastroenterology 1996, 111, 462. 8690213 10.1053/gast.1996.v111.pm8690213

[41] M. Ballegeer , C. Libert , J. Gastroenterol. Hepatol. Res. 2016, 1.

[42] B. O. Zhou , R. Yue , M. M. Murphy , J. G. Peyer , S. J. Morrison , Cell Stem Cell 2014, 15, 154. 24953181 10.1016/j.stem.2014.06.008 PMC4127103

[43] Y.‐T. Yen , M. Chien , P.‐Y. Wu , S.‐C. Hung , Commun. Biol. 2021, 4, 658. 34079065 10.1038/s42003-021-02175-1 PMC8172534

[44] K. M. Pineault , J. Y. Song , K. M. Kozloff , D. Lucas , D. M. Wellik , Nat. Commun. 2019, 10, 3168. 31320650 10.1038/s41467-019-11100-4 PMC6639390

[45] M. Decker , L. Martinez‐Morentin , G. Wang , Y. Lee , Q. Liu , J. Leslie , L. Ding , Nat. Cell Biol. 2017, 19, 677. 28481328 10.1038/ncb3530 PMC5801040

[46] Y. Chen , L.‐X. Xiang , J.‐Z. Shao , R.‐L. Pan , Y.‐X. Wang , X.‐J. Dong , G.‐R. Zhang , J. Cell. Mol. Med. 2010, 14, 1494. 19780871 10.1111/j.1582-4934.2009.00912.x PMC3829016

[47] Y. Tang , X. Wu , W. Lei , L. Pang , C. Wan , Z. Shi , L. Zhao , T. R. Nagy , X. Peng , J. Hu , X. Feng , W. Van Hul , M. Wan , X. Cao , Nat. Med. 2009, 15, 757. 19584867 10.1038/nm.1979 PMC2727637

[48] Y. Liu , X. Yang , Y. Jing , S. Zhang , C. Zong , J. Jiang , K. Sun , R. Li , L. Gao , X. Zhao , D. Wu , Y. Shi , Z. Han , L. Wei , Sci. Rep. 2015, 5, 17762. 26643997 10.1038/srep17762 PMC4672342

[49] G. Ren , L. Zhang , X. Zhao , G. Xu , Y. Zhang , A. I. Roberts , R. C. Zhao , Y. Shi , Cell Stem Cell 2008, 2, 141. 18371435 10.1016/j.stem.2007.11.014

[50] F. Cottini , T. Hideshima , C. Xu , M. Sattler , M. Dori , L. Agnelli , E. Ten Hacken , M. T. Bertilaccio , E. Antonini , A. Neri , M. Ponzoni , M. Marcatti , P. G. Richardson , R. Carrasco , A. C. Kimmelman , K.‐K. Wong , F. Caligaris‐Cappio , G. Blandino , W. M. Kuehl , K. C. Anderson , G. Tonon , Nat. Med. 2014, 20, 599. 24813251 10.1038/nm.3562 PMC4057660

[51] F. Cottini , K. C. Anderson , G. Tonon , Mol. Cell Oncol. 2014, 1, e970055. 27308358 10.4161/23723548.2014.970055 PMC4904900

[52] Y. Matsuzaki , Y. Mabuchi , H. Okano , Cell Stem Cell 2014, 15, 112. 25105573 10.1016/j.stem.2014.07.001

[53] X. Han , Q. Yang , L. Lin , C. Xu , C. Zheng , X. Chen , Y. Han , M. Li , W. Cao , K. Cao , Q. Chen , G. Xu , Y. Zhang , J. Zhang , R. J. Schneider , Y. Qian , Y. Wang , G. Brewer , Y. Shi , Cell Death Differ. 2014, 21, 1758. 25034782 10.1038/cdd.2014.85 PMC4211372

[54] H. Kleinert , A. Pautz , K. Linker , P. M. Schwarz , Eur. J. Pharmacol. 2004, 500, 255. 15464038 10.1016/j.ejphar.2004.07.030

[55] M.‐K. Kim , J.‐W. Jang , S.‐C. Bae , BMB Rep. 2018, 51, 126. 29366442 10.5483/BMBRep.2018.51.3.015 PMC5882219

[56] Z. Shi , F. He , M. Chen , L. Hua , W. Wang , S. Jiao , Z. Zhou , Oncogene 2017, 36, 4362. 28368398 10.1038/onc.2017.24

[57] B. Zhao , X. Ye , J. Yu , L. Li , W. Li , S. Li , J. Yu , J. D. Lin , C.‐Y. Wang , A. M. Chinnaiyan , Z.‐C. Lai , K.‐L. Guan , Genes Dev. 2008, 22, 1962. 18579750 10.1101/gad.1664408 PMC2492741

[58] P. Agarwal , S. Isringhausen , H. Li , A. J. Paterson , J. He , Á. Gomariz , T. Nagasawa , C. Nombela‐Arrieta , R. Bhatia , Cell Stem Cell 2019, 24, 769. 30905620 10.1016/j.stem.2019.02.018 PMC6499704

[59] S. Méndez‐Ferrer , T. V. Michurina , F. Ferraro , A. R. Mazloom , B. D. Macarthur , S. A. Lira , D. T. Scadden , A. Ma'ayan , G. N. Enikolopov , P. S. Frenette , Nature 2010, 466, 829. 20703299 10.1038/nature09262 PMC3146551

[60] L. Xie , X. Zeng , J. Hu , Q. Chen , Stem Cells Int. 2015, 762098. 26236348 10.1155/2015/762098 PMC4506912

[61] S. Wangler , U. Menzel , Z. Li , J. Ma , S. Hoppe , L. M. Benneker , M. Alini , S. Grad , M. Peroglio , Osteoarthritis Cartilage 2019, 27, 1094. 31002939 10.1016/j.joca.2019.04.002

[62] C. Zhang , X. Han , J. Liu , L. Chen , Y. Lei , K. Chen , J. Si , T.‐Y. Wang , H. Zhou , X. Zhao , X. Zhang , Y. An , Y. Li , Q.‐F. Wang , Genomics Proteomics Bioinformatics 2022, 20, 70. 35123072 10.1016/j.gpb.2022.01.005 PMC9510874

[63] U. Rashid , A. Yousaf , M. Yaqoob , E. Saba , M. Moaeen‐Ud‐Din , S. Waseem , S. K. Becker , G. Sponder , J. R. Aschenbach , M. A. Sandhu , BMC Vet. Res. 2021, 17, 388. 34922529 10.1186/s12917-021-03100-8 PMC8684202

[64] S. Nishimura , I. Manabe , M. Nagasaki , K. Eto , H. Yamashita , M. Ohsugi , M. Otsu , K. Hara , K. Ueki , S. Sugiura , K. Yoshimura , T. Kadowaki , R. Nagai , Nat. Med. 2009, 15, 914. 19633658 10.1038/nm.1964

[65] J. A. Wiley , A. Cerwenka , J. R. Harkema , R. W. Dutton , A. G. Harmsen , Am. J. Pathol. 2001, 158, 119. 11141485 10.1016/s0002-9440(10)63950-8 PMC1850251

[66] L. Chapat , K. Chemin , B. Dubois , R. Bourdet‐Sicard , D. Kaiserlian , Eur. J. Immunol. 2004, 34, 2520. 15307184 10.1002/eji.200425139

[67] H. E. Thomas , J. A. Trapani , T. W. H. Kay , Cell Death Differ. 2010, 17, 577. 19927156 10.1038/cdd.2009.165

[68] M. Leist , A. Wendel , J. Hepatol. 1996, 25, 948. 9007725 10.1016/s0168-8278(96)80301-1

[69] H.‐X. Wang , World J. Gastroenterol. 2012, 18, 119. 22253517 10.3748/wjg.v18.i2.119 PMC3257438

[70] T. Miyagi , T. Takehara , T. Tatsumi , T. Suzuki , M. Jinushi , Y. Kanazawa , N. Hiramatsu , T. Kanto , S. Tsuji , M. Hori , N. Hayashi , Hepatology 2004, 40, 1190. 15486936 10.1002/hep.20447

[71] C. S. Bonder , M. N. Ajuebor , L. D. Zbytnuik , P. Kubes , M. G. Swain , J. Immunol. 2004, 172, 45. 14688308 10.4049/jimmunol.172.1.45

[72] D. L. Weinbaum , J. A. Sullivan , G. L. Mandell , Nature 1980, 286, 725. 7412858 10.1038/286725a0

[73] L. Fu , Y. Hu , M. Song , Z. Liu , W. Zhang , F.‐X. Yu , J. Wu , S. Wang , J. C. Izpisua Belmonte , P. Chan , J. Qu , F. Tang , G.‐H. Liu , PLoS Biol. 2019, 17, e3000201. 30933975 10.1371/journal.pbio.3000201 PMC6459557

[74] G. Loforese , T. Malinka , A. Keogh , F. Baier , C. Simillion , M. Montani , T. D. Halazonetis , D. Candinas , D. Stroka , EMBO Mol. Med. 2017, 9, 46. 27940445 10.15252/emmm.201506089 PMC5210079

[75] H. Jin , N. Lian , F. Zhang , M. Bian , X. Chen , C. Zhang , Y. Jia , C. Lu , M. Hao , S. Yao , J. Shao , L. Wu , A. Chen , S. Zheng , Eur. J. Pharm. Sci. 2017, 96, 323. 27717875 10.1016/j.ejps.2016.10.002

[76] D. Elster , B. Von Eyss , Mech Ageing Dev 2020, 189, 111280. 32512018 10.1016/j.mad.2020.111280

[77] L. Han , Q. Long , S. Li , Q. Xu , B. Zhang , X. Dou , M. Qian , Y. Jiramongkol , J. Guo , L. Cao , Y. E. Chin , E. W.‐F. Lam , J. Jiang , Y. Sun , Cancer Res. 2020, 80, 3383. 32366480 10.1158/0008-5472.CAN-20-0506 PMC7611217

[78] Y. Horimoto , U. M. Polanska , Y. Takahashi , A. Orimo , Cell Adh. Migr. 2012, 6, 193. 22568980 10.4161/cam.20631 PMC3427234

[79] X. G. Yu , M. Lichterfeld , Cell Cycle 2012, 11, 4097.23075495

[80] P. Callihan , J. Mumaw , D. W. Machacek , S. L. Stice , S. B. Hooks , Pharmacol. Ther. 2011, 129, 290. 21073897 10.1016/j.pharmthera.2010.10.007

[81] A. S. Hauser , M. M. Attwood , M. Rask‐Andersen , H. B. Schiöth , D. E. Gloriam , Nat. Rev. Drug Discov. 2017, 16, 829. 29075003 10.1038/nrd.2017.178 PMC6882681

[82] P. Santos‐Otte , H. Leysen , J. Van Gastel , J. O. Hendrickx , B. Martin , S. Maudsley , Comput. Struct. Biotechnol. J. 2019, 17, 1265. 31921393 10.1016/j.csbj.2019.08.005 PMC6944711

[83] A. Aihara , T. Iwawaki , N. Abe‐Fukasawa , K. Otsuka , K. Saruhashi , T. Mikashima , T. Nishino , J. Biol. Chem. 2022, 298, 101779. 35231442 10.1016/j.jbc.2022.101779 PMC8988011

[84] R. Johnson , G. Halder , Nat. Rev. Drug Discov. 2014, 13, 63. 24336504 10.1038/nrd4161 PMC4167640

[85] T. Stuart , A. Butler , P. Hoffman , C. Hafemeister , E. Papalexi , W. M. 3rd Mauck , Y. Hao , M. Stoeckius , P. Smibert , R. Satija , Compr. Integr. Single‐Cell Data Cell 2019, 177, 1888. 10.1016/j.cell.2019.05.031 PMC6687398 31178118

[86] D. Aran , A. P. Looney , L. Liu , E. Wu , V. Fong , A. Hsu , S. Chak , R. P. Naikawadi , P. J. Wolters , A. R. Abate , A. J. Butte , M. Bhattacharya , Nat. Immunol. 2019, 20, 163. 30643263 10.1038/s41590-018-0276-y PMC6340744

[87] C. Zhang , L. Lei , X. Yang , K. Ma , H. Zheng , Y. Su , A. Jiao , X. Wang , H. Liu , Y. Zou , L. Shi , X. Zhou , C. Sun , Y. Hou , Z. Xiao , L. Zhang , B. Zhang , J. Immunother. Cancer 2021, 9, e002809. 34642245 10.1136/jitc-2021-002809 PMC8513495

[88] H. Wang , X.‐X. Feng , P. Han , Y. Lei , Y.‐J. Xia , D. Tian , W. Yan , Mol. Med. Rep. 2019, 20, 4883. 31638166 10.3892/mmr.2019.10750 PMC6854585

[89] I. Mohar , K. J. Brempelis , S. A. Murray , M. R. Ebrahimkhani , I. N. Crispe , Methods Mol. Biol. 2015, 1325, 3. 26450375 10.1007/978-1-4939-2815-6_1

[90] H. Zhu , Z.‐K. Guo , X.‐X. Jiang , H. Li , X.‐Y. Wang , H‐Y. Yao , Y. Zhang , N. Mao , Nat. Protoc. 2010, 5, 550. 20203670 10.1038/nprot.2009.238

[91] Y.‐Y. Jing , Z.‐P. Han , K. Sun , S.‐S. Zhang , J. Hou , Y. Liu , R. Li , L. Gao , X. Zhao , Q.‐D. Zhao , M.‐C. Wu , L.‐X. Wei , BMC Med. 2012, 10, 98. 22938142 10.1186/1741-7015-10-98 PMC3482562

