# Correction to “Hippo Pathway Activation in Aged Mesenchymal Stem Cells Contributes to the Dysregulation of Hepatic Inflammation in Aged Mice”

**DOI:** 10.1002/advs.202509745

**Published:** 2025-06-18

Yang, et al. Hippo Pathway Activation in Aged Mesenchymal Stem Cells Contributes to the Dysregulation of Hepatic Inflammation in Aged Mice. Advanced Science.2023 Sep;10 (27):e2300424.

https://doi.org/10.1002/advs.202300424


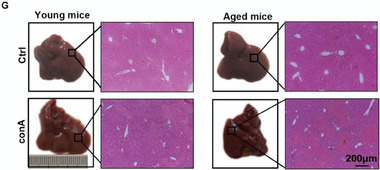


In Figure 1G, the figure shown above was incorrect.

This should have read as follows:


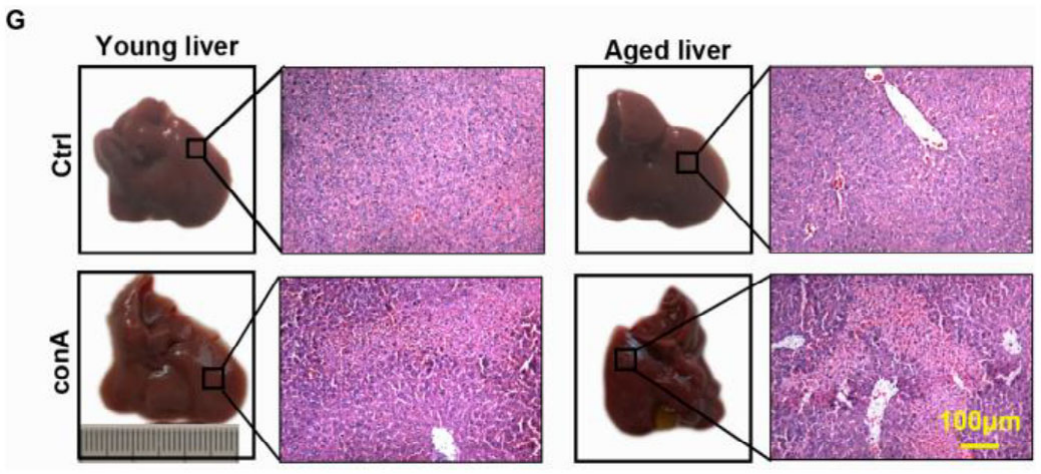



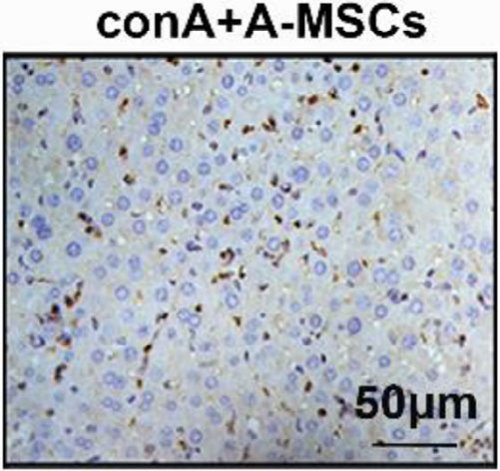


In Figure 2H, the image of conA+A‐MSCs shown above was incorrect.

This should have read as shown below:


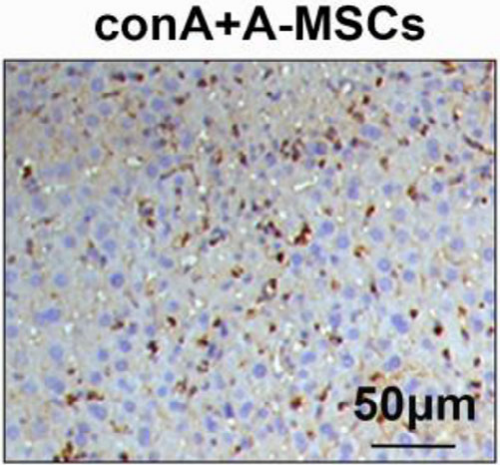



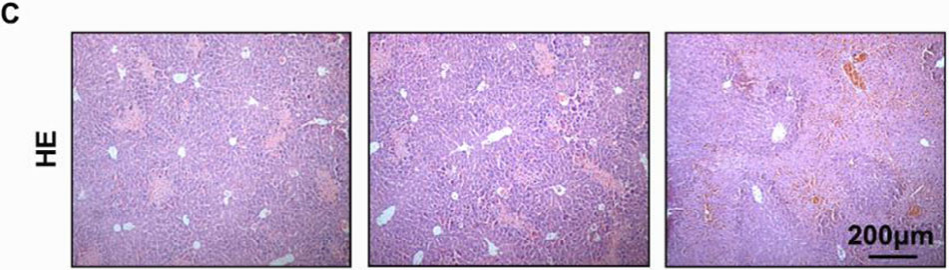


In Figure 4C, the figure shown above was incorrect.

This should have read as follows:


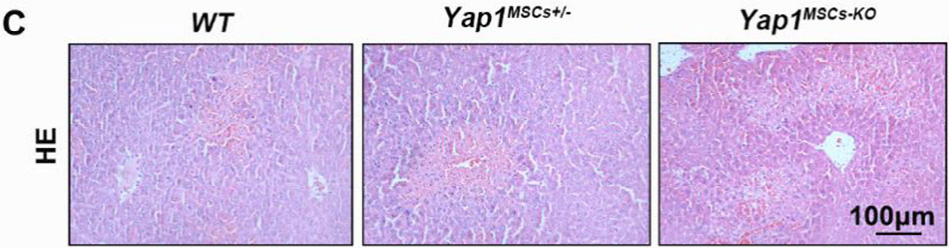


After a thorough review of all raw data and original experimental records, we have confirmed that these corrections do not affect the study's conclusions.

We apologize for the errors.

