# Supplementary material for: Hippo Pathway Activation in Aged Mesenchymal Stem Cells Contributes to the Dysregulation of Hepatic Inflammation in Aged Mice
Source: Adv Sci (Weinh). 2023 Aug 6;10(27):2300424. doi: 10.1002/advs.202300424 (PMC10520691; doi:10.1002/advs.202300424)
Supplement: Supplementary file 1 — Supporting Information [file ADVS-10-2300424-s001.pdf]

ADVANCED SCIENCE
Open Access

Supporting Information

for *Adv. Sci.*, DOI 10.1002/advs.202300424

Hippo Pathway Activation in Aged Mesenchymal Stem Cells Contributes to the Dysregulation of Hepatic Inflammation in Aged Mice

Xue Yang, Chen Zong, Chao Feng, Cangang Zhang, Artem Smirnov, Gangqi Sun, Changchun Shao, Luyao Zhang, Xiaojuan Hou, Wenting Liu, Yan Meng, Liying Zhang, Changshun Shao, Lixin Wei, Gerry Melino* and Yufang Shi**

Supplementary Table 1. The mouse gene primers for Real-time PCR

Gene	Primers
Yap1	Forward 5’-ACCCTCGTTTTGCCATGAAC-3’<br>Reverse 5’-TGTGCTGGGATTGATATTCCGTA-3’
Areg	Forward 5’-GGTCTTAGGCTCAGGCCATTA-3’<br>Reverse 5’-CGCTTATGGTGGAAACCTCTC-3’
Ccnb1	Forward 5’-AAGGTGCCTGTGTGTGAACC-3’<br>Reverse 5’-GTCAGCCCCATCATCTGCG-3’
Birc5	Forward 5’-GAGGCTGGCTTCATCCACTG-3’<br>Reverse 5’-CTTTTTGCTTGTTGTTGGTCTCC-3’
Cyr61	Forward 5’-CTGCGCTAAACAACTCAACGA-3’<br>Reverse 5’-GCAGATCCCTTTCAGAGCGG-3’
Foxm1	Forward 5’-CTGATTCTCAAAAGACGGAGGC-3’<br>Reverse 5’-TTGATAATCTTGATTCCGGCTGG-3’
Nos2	Forward 5’- ACATCGACCCGTCCACAGTAT-3’<br>Reverse 5’- CAGAGGGGTAGGCTTGTCTC-3’
Tnfaip6	Forward 5’-GTGAGCGATGGGATGCCTATT-3’<br>Reverse 5’-AGCCGAATGTGCCAGTAGC-3’
Tgfβ1	Forward 5’-CTCCCGTGGCTTCTAGTGC-3’<br>Reverse 5’-GCCTTAGTTTGGACAGGATCTG-3’
Il-10	Forward 5’-GCTCTTACTGACTGGCATGAG-3’<br>Reverse 5’-CGCAGCTCTAGGAGCATGTG-3’
Stat1	Forward 5’- TCACAGTGGTTCGAGCTTCAG-3’<br>Reverse 5’- GCAAACGAGACATCATAGGCA-3’

Supplementary Figure and Figure legends.

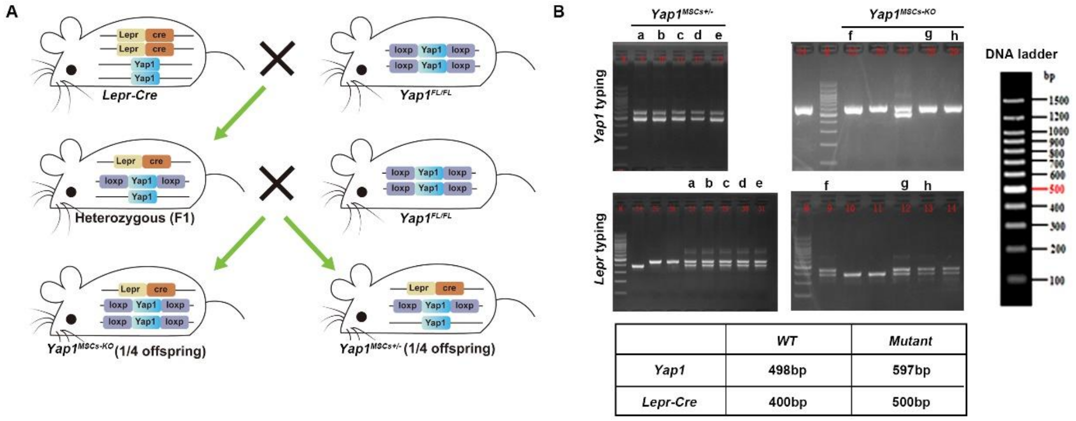

Figure S1. Generation of *Yap1*$^{MSCs\text{-}KO}$ mice and subsequent genotyping analysis.

A, *Yap1*$^{FL/FL}$ and *Lepr*-*cre* mice were used to obtain heterozygous *Yap1*$^{MSCs+/-}$ and homozygous *Yap1*$^{MSCs\text{-}KO}$ mice. B, Genotyping of *Yap1* and *Lepr* mice were performed by PCR with the indicated primers. a, b, c, d, e: *Yap1*$^{MSCs+/-}$; f, g, h: *Yap1*$^{MSCs\text{-}KO}$.

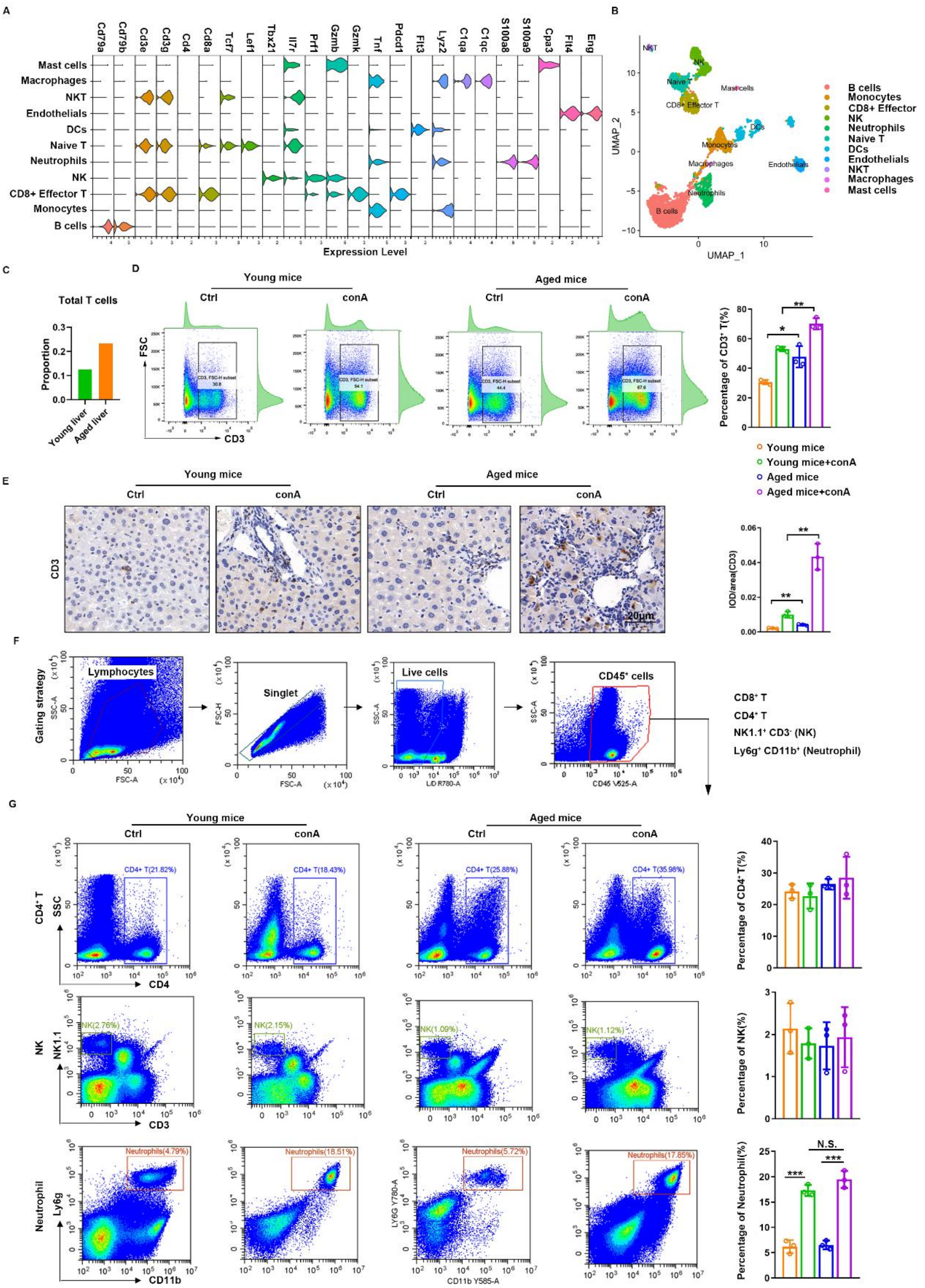

Figure S2. More CD8^{+} T cells were activated in aged liver. Previous scRNA-seq data of young and aged mice were re-analyzed (A-C). A, Violin plot of immune cell markers of young and aged mice liver. B, Identification and annotation of different clusters of cell types in young and aged mice liver. C, Proportion of Total T cells including CD8^{+} effector T cells, naïve T cells and NKT

cells in young and aged liver. D, $CD3^+$ T cells in liver were identified by FACS and quantified. E, IHC staining and quantification of $CD3^+$ T cells in the liver. F, Gating strategy of different immune cell types. G, $CD4^+$ T cells, NK cells and neutrophils were analyzed in young and aged mice before and after conA treatment by FACS and then quantified. Data were analyzed using two-tailed unpaired Student t-test. *$p<0.05$, **$p<0.01$, ***$p<0.001$. N.S. $p\geq0.05$. Graphs showed mean ± S.D. N=3, the experiments were repeated for three times.

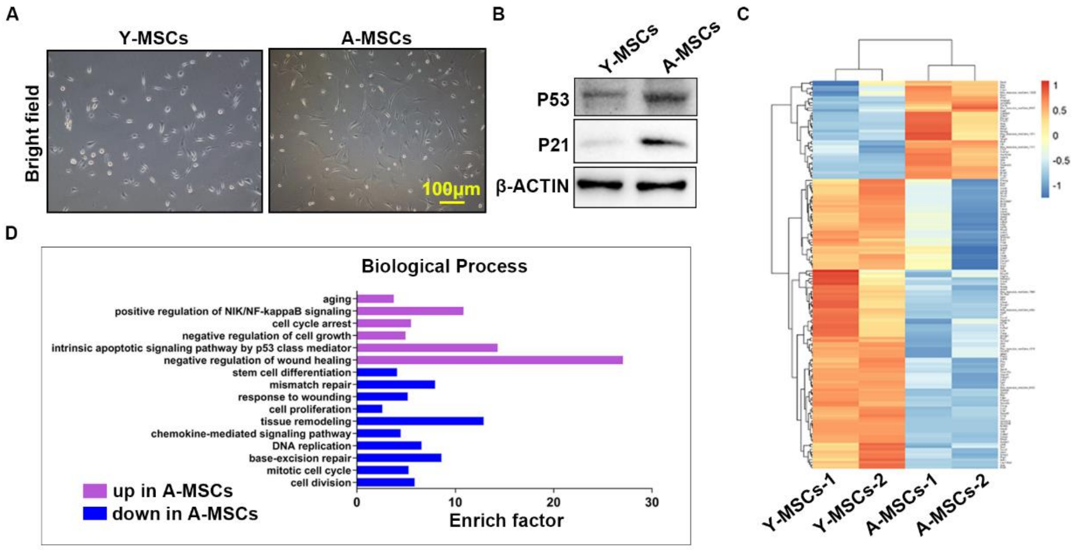

Figure S3. Characteristics and mRNA expression pattern of young and aged MSCs.

A, Morphology of young and aged MSCs under bright field. B, Expression levels of P53 and P21 in young and aged MSCs were detected by western blotting. β-ACTIN was used as the internal control. C, Heatmap of transcriptome data showing differences of gene expression levels in young and aged MSCs. D, Upregulated and downregulated biological processes in aged MSCs. Y-MSCs, young MSCs; A-MSCs, aged MSCs.

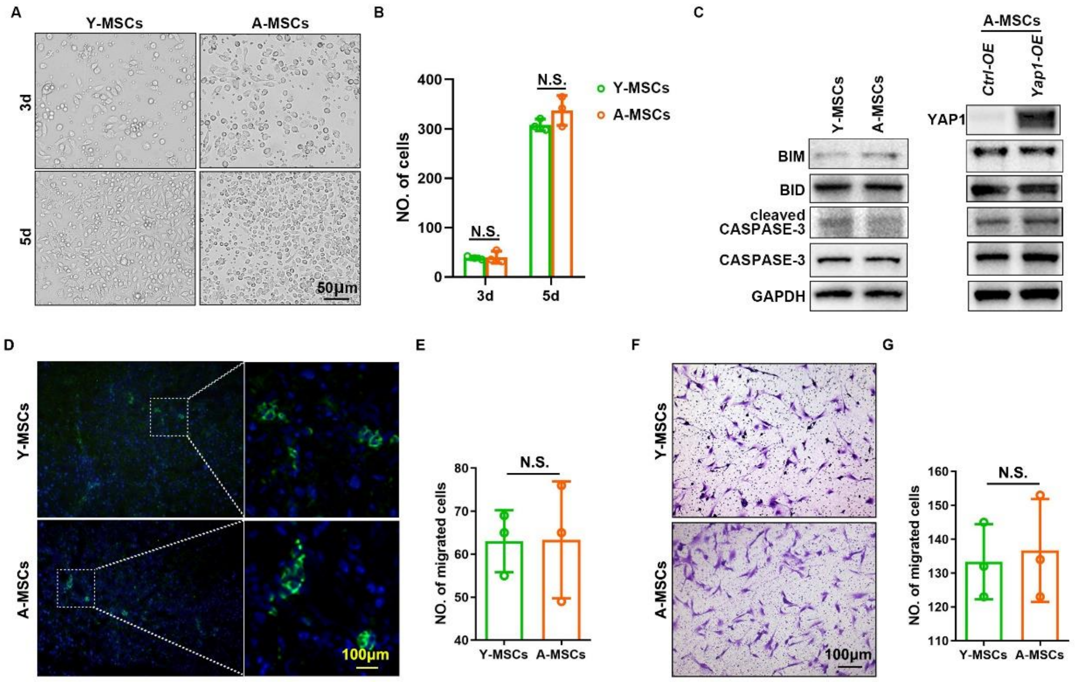

Figure S4. Acquisition, apoptosis detection, local recruitment and migration of young and aged MSCs. A, B, Observation and quantification of young and aged MSCs at various time points post-isolation. C, Apoptosis-associated proteins were detected in both young and aged MSCs, as well as in *Yap1*-overexpressing aged MSCs. GAPDH was used as internal control. D, E, EGFP-labeled young and aged MSCs were injected into mice with liver injury induced by conA. Immunofluorescence staining against EGFP was performed of frozen liver sections at 8h post-transplantation and $EGFP^{+}$ cells were subsequently quantified. F, G, Migration ability of young and aged MSCs was tested by transwell assay *in vitro*. Migrated cells were stained with crystal violet and quantified. Y-MSCs, young MSCs; A-MSCs, aged MSCs. Data were analyzed using two-tailed unpaired Student t-test. N.S. $p \geq 0.05$. Graphs showed mean ± S.D. N=3 or 4, the experiments were repeated for three times.

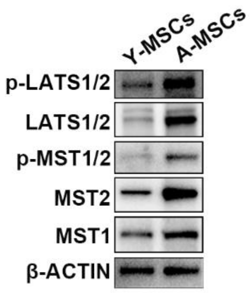

Figure S5. Western blotting analysis of kinases in the Hippo signaling pathway in young and aged MSCs. β-ACTIN was used as the internal control. Y-MSCs, young MSCs; A-MSCs, aged MSCs.

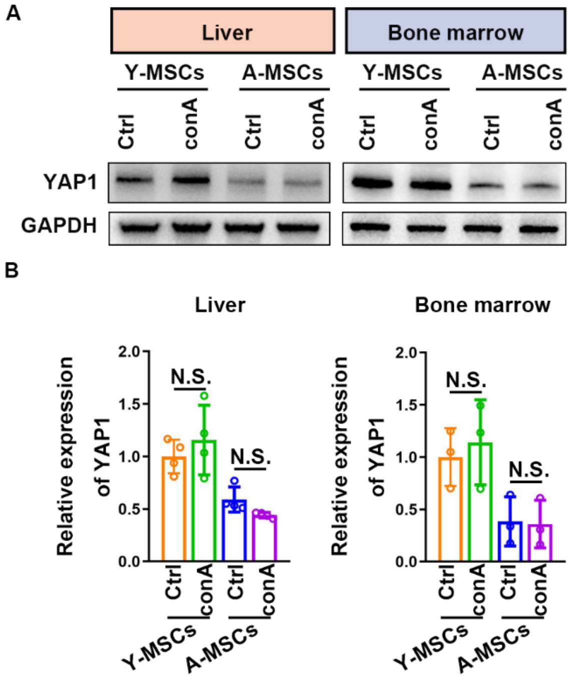

Figure S6. The impact of conA on YAP1 expression *in vivo* in both young and aged MSCs. A, YAP1 expression was examined by western blotting assay in liver and bone marrow MSCs from both young and aged individuals, both before and after induction with conA. GAPDH was used as the internal control. B, YAP1 expression relative to GAPDH was quantified according to liver MSCs from young mice without conA treatment. Y-MSCs, young MSCs; A-MSCs, aged MSCs. Data were analyzed using two-tailed unpaired Student t-test. N.S. $p \geq 0.05$. Graphs showed mean ± S.D. N=3 or 4, the experiments were repeated for three times.

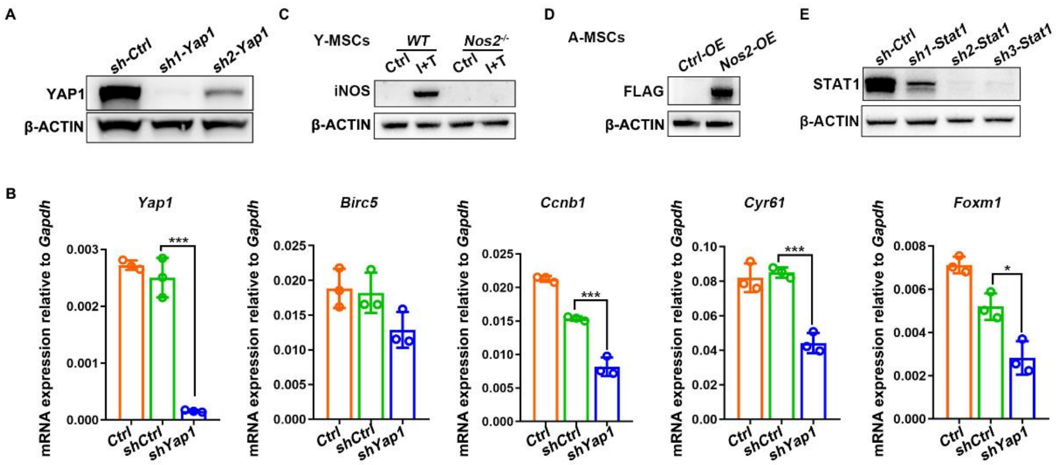

Figure S7. Verification of the efficiency of various adenoviruses and gene knockout mice.

A, Young MSCs were transfected by *sh1-Yap1* and *sh2-Yap1* adenovirus. 24 h later, YAP1 expression was detected by western blotting. β-ACTIN was used as the internal control. B, The mRNA expression levels of *Yap1* and its target genes were measured by RT-PCR. C, *WT* and *Nos2*$^{-/-}$ young MSCs were treated with I+T for 24h, iNOS was detected by western blotting. β-actin was used as the internal control. D, Aged MSCs were transfected with *Nos2* overexpression adenovirus. The fusion protein FLAG-iNOS expression was detected by using anti-FLAG antibody by western blotting. β-ACTIN was used as the internal control. E, Young MSCs were transfected by *sh1-Stat1*, *sh2-Stat1* and *sh3-Stat1* adenovirus. 24 h later, STAT1 expression was detected by western blotting. β-ACTIN was used as the internal control. Data were analyzed using two-tailed unpaired Student t-test. *p<0.05, ***p<0.001. Graphs showed mean ± S.D. N=3, the experiments were repeated for three times.

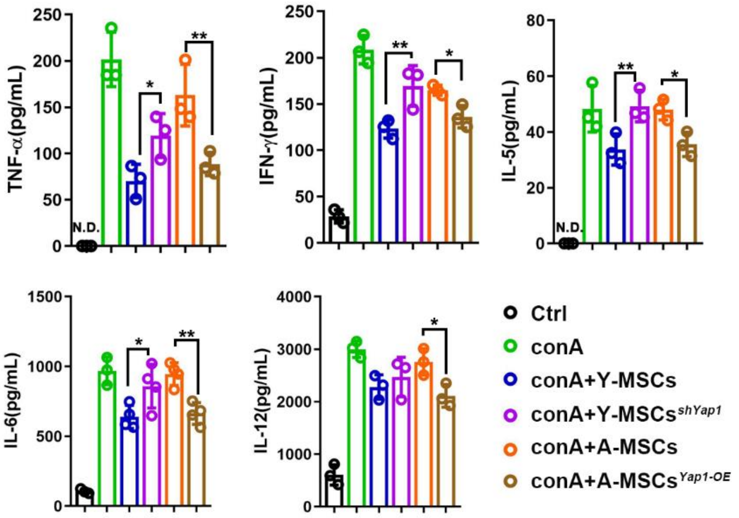

Figure S8. Serum levels of inflammatory factors were assessed in conA-treated mice following injection with young and aged MSCs, both with and without manipulation of *Yap1* expression. The concentrations of inflammatory factors were detected by Bio-plex assay. Y-MSCs, young MSCs; A-MSCs, aged MSCs. N.D., not detected. Data were analyzed using one-way ANOVA test. *p<0.05, **p<0.01. Graphs showed mean ± S.D. N=3 or 4, the experiments were repeated for three times.

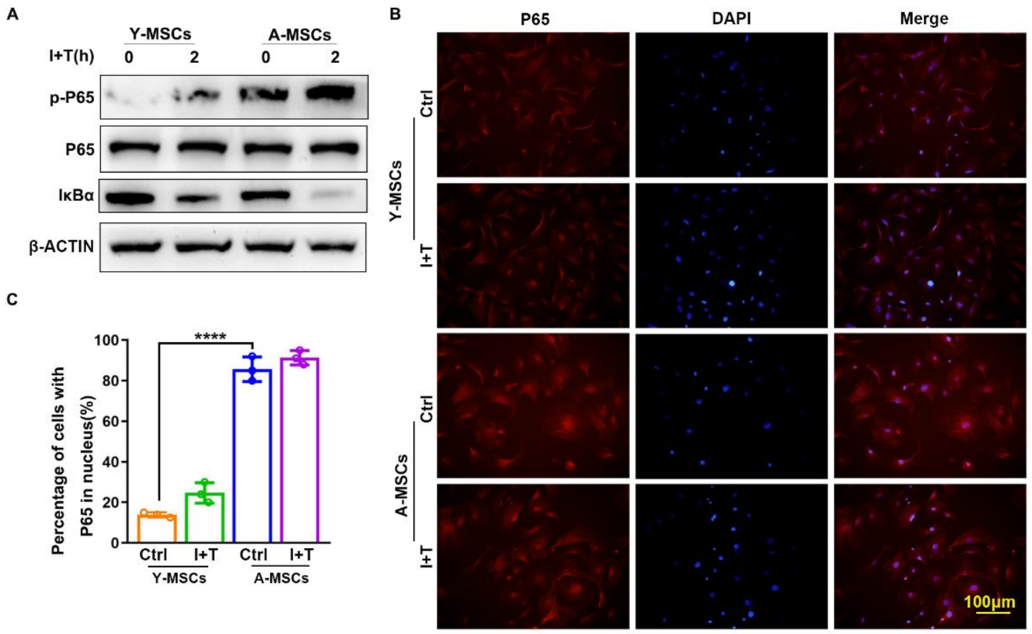

Figure S9. Analysis of the NF-κB signaling pathway in young and aged MSCs. A, Young and aged MSCs were treated with I+T for 0 h and 1 h. Expression of P65 and p-P65 were detected by western blotting. β-ACTIN was used as the internal control. B, Immunofluorescence staining of P65 was performed to detect its translocation into the nucleus after I+T treatment for 1 h. C, The proportion of cells exhibiting nuclear localization of P65. Y-MSCs, young MSCs; A-MSCs, aged MSCs. Data were analyzed using two-tailed unpaired Student t-test. ****p<0.0001. Graph showed mean ± S.D. N=3, the experiments were repeated for three times.

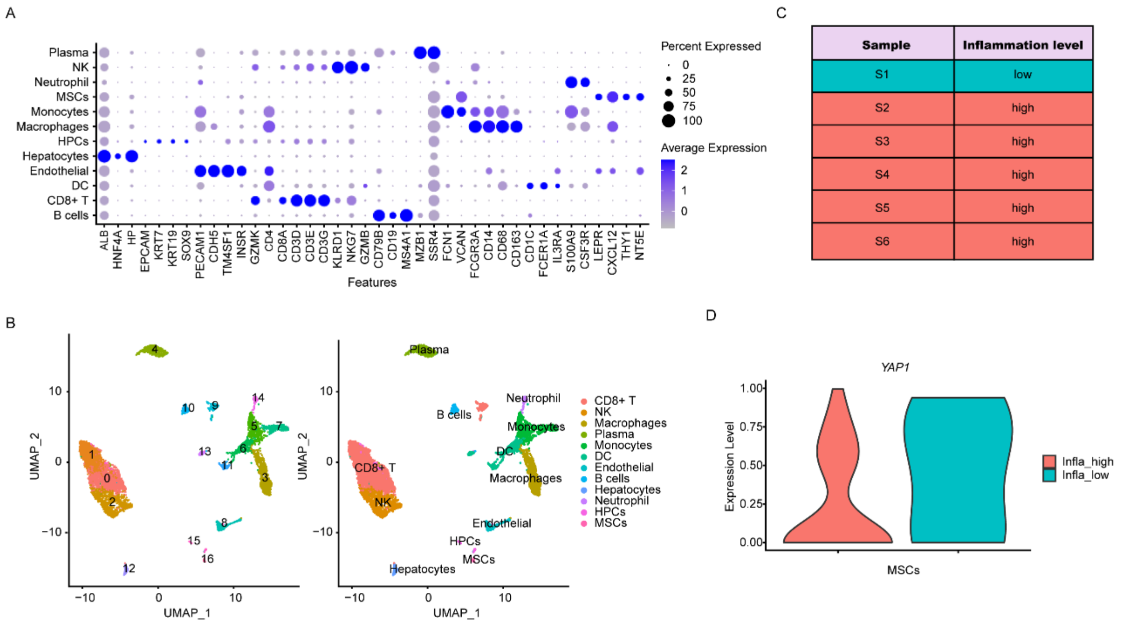

Sample	Inflammation level
S1	low
S2	high
S3	high
S4	high
S5	high
S6	high

Figure S10. The expression level of *YAP1* was found to be higher in MSCs from samples with lower levels of inflammation. Previous scRNA-seq data of human liver fibrosis was re-analyzed. A, Dotplot of cell markers for different cell types in the human liver. B, Identification and annotation of different cell types in the human liver. C, The information level of the samples. D, Vlnplot showed expression of *YAP1* in MSCs from samples with different levels of inflammation.

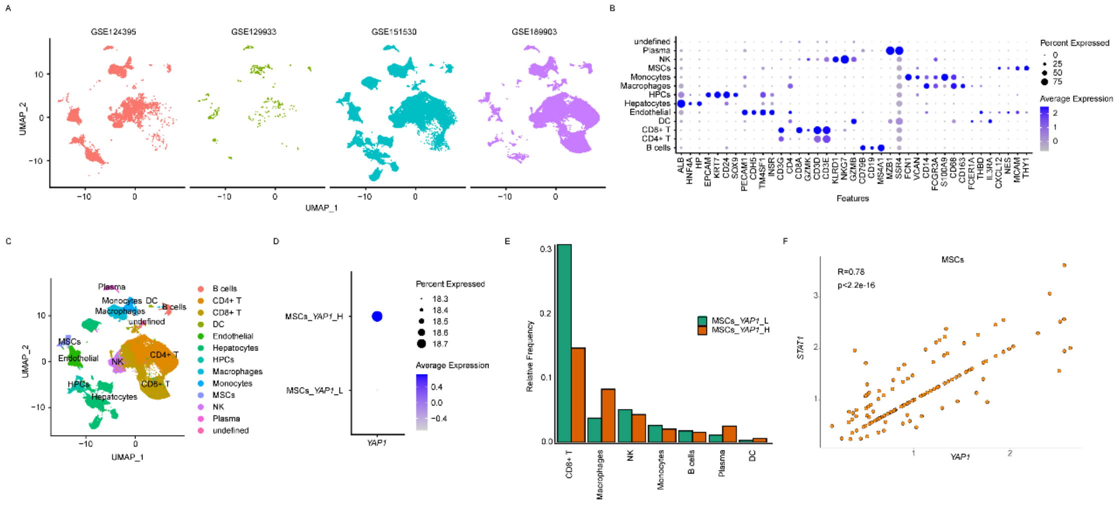

Figure S11. Correlation between *YAP1* expression in MSCs and $CD8^{+}$ T cell infiltration, as well as *STAT1* expression in human liver diseases. Four scRNA-seq datasets of human liver diseases were integrated together and re-analyzed. A, Presentation of the integration of the four datasets by UMAP. B, Dotplot of cell markers for different cell types in the human liver. C, Identification and annotation of different cell types in the human liver. D, Differential expression levels of *YAP1* in MSCs. E, The ratio of various immune cell types in samples with different levels of *YAP1* expression in MSCs. F, Correlation between *YAP1* and *STAT1* in MSCs of human liver was analysed using ggplot2 package.
